# Amphiphilic Zeolitic Imidazolate Framework for Improved CO_2_ Separation in PIM‐1 Mixed Matrix Membranes

**DOI:** 10.1002/anie.202420879

**Published:** 2025-04-14

**Authors:** Marta Pérez‐Miana, José Miguel Luque‐Alled, Álvaro Mayoral, Íñigo Martínez‐Visus, Andrew B. Foster, Peter M. Budd, Joaquín Coronas

**Affiliations:** ^1^ Nanoscience and Materials Institute of Aragon (INMA) CSIC‐Universidad de Zaragoza Mariano Esquillor St. Zaragoza 50018 Spain; ^2^ Department of Chemical and Environmental Engineering Universidad de Zaragoza María de Luna, 3 St. Zaragoza 50018 Spain; ^3^ Department of Chemistry School of Natural Sciences The University of Manchester Manchester M13 9PL UK

**Keywords:** Amphiphilicity, CO_2_ capture, Mixed matrix membrane, Polymer of intrinsic microporosity‐1, Zeolitic imidazolate framework‐94

## Abstract

This study aims to enhance the compatibility between filler and polymer in mixed matrix membranes (MMMs), addressing an important challenge in membrane development. ZIF‐94, known for its affinity to CO_2_, was partially modified with 2‐undecylimidazolate (umIm) through the solvent‐assisted ligand exchange (SALE) method to improve its compatibility with the prototypical polymer of intrinsic microporosity PIM‐1. The modified ZIF‐94 (ZIF‐94‐umIm) can be considered as an amphiphilic MOF with both hydrophilic and hydrophobic moieties, while maintaining a considerably high CO_2_ adsorption capacity (2.34 mmol g^−1^ at 90 kPa and 0 °C). Gas separation experiments were performed using mixed gas compositions of 15/85 CO_2_/N_2_ at 3 bar and 35 °C. The resulting MMM with a 5 wt.% loading exhibited an enhanced CO_2_ separation performance, with ca. 70% and 10% increases in CO_2_ permeability (8900 Barrer) and CO_2_/N_2_ selectivity (20.2), respectively, compared to pristine PIM‐1 membranes. In addition, thin film nanocomposite membranes were prepared showing a 23.5 CO_2_/N_2_ selectivity at 2350 GPU of CO_2_. This modification strategy shows a great potential for improving the CO_2_ capture technologies, highlighting the potential of tailoring MOF fillers for advanced membrane materials in gas separation applications.

## Graphical abstract

An improvement in compatibility between filler ZIF‐94 and polymer PIM‐1 in mixed matrix membranes (MMMs) was achieved through ligand exchange with undecylimidazole (umIm). The modified ZIF‐94 (ZIF‐94‐umIm) presents amphiphilic character and high CO_2_ adsorption (2.34 mmol g^−1^ at 90 kPa and 0 °C). The MMM with 5 wt.% loading showed a 70% increase in CO_2_ permeability (8900 Barrer) and 10% higher CO_2_/N_2_ selectivity (20.2) compared to pure PIM‐1 membranes.

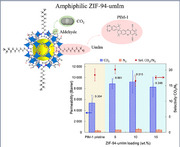

## Introduction

Gas separation technologies play an essential role in diverse industrial processes, extending from carbon dioxide (CO_2_) capture to the production of high‐purity gases for various applications. Membrane‐based separation processes offer several advantages over conventional technologies due to their compact system design, operational simplicity, reduced energy consumption, smaller footprint, and scalability.^[^ ^1^, ^2^ ^]^ Membranes have shown great potential for remediating CO_2_ emissions by separating it from gaseous mixtures, such as flue gas (CO_2_/N_2_ separations) and natural gas (CO_2_/CH_4_).^[^ ^3^, ^4^ ^]^ Polymeric membranes are preferred due to their cost‐effectiveness, ease of production, and ability to be tailored to specific requirements. Industrially used polymers for CO_2_/N_2_ separations provide adequate selectivity and stable performance over time.^[^ ^5^ ^]^ Along the last few decades, several polymers with advanced performance at a laboratory scale have been engineered. Among these, polymers of intrinsic microporosity (PIMs), and particularly PIM‐1, stand out for their large free volume, intrinsic microporosity, and exceptional CO_2_ permeability.^[^ ^6^ ^]^ Its rigid and contorted structure prevents the efficient packing of polymer chains, resulting in the formation of micropores (<2 nm) that exhibit gas sieving properties. Although PIM‐1 demonstrates exceptional gas separation performance for various gas pairs, such as O_2_/N_2_, CO_2_/CH_4_, and CO_2_/N_2_, it faces several challenges for its commercialization. These include insufficient gas pair selectivity required in some industrial processes, stability against physical aging and plasticization, scalability of polymer production, and reproducible membrane fabrication.

Mixed matrix membranes (MMMs) are formed by the integration of nanofillers into a polymeric membrane as a viable solution to overcome the weaknesses of both polymeric and inorganic membranes.^[^ ^5^ ^]^ Metal‐organic frameworks (MOFs) have garnered attention as potential filler materials for the preparation of MMMs due to their unique properties, including crystallinity, tailorable chemical composition and structure, high porosity, tunable pore size, compatibility, and affordability. MOFs based on imidazolate ligands, known as ZIFs (zeolitic imidazolate frameworks), are particularly promising for membrane separation applications due to their high surface area, large porosity, and affordable scalability.^[^ ^7^ ^]^ ZIF‐94, also referred to as (Substituted Imidazolate Material‐1),^[^ ^8^ ^]^ (SIM‐1) presents a SOD‐type structure composed of zinc metal ions (Zn^2+^) coordinated with a ligand functionalized with an aldehyde group, the 4‐methyl‐5‐imidazolecarboxaldehyde (mImca) ligand.^[^ ^9^ ^]^ This MOF exhibits cavities measuring 9.6 Å accessible through 2.6 Å pore openings (slightly smaller than those of ZIF‐8). ZIF‐94 exhibits a hydrophilic nature and significantly high CO_2_ adsorption capacity (CO_2_ uptake of 3.3 mmol g^−1^ at 1 bar). This is attributed to the presence of polar functional groups within its ligand structure,^[^ ^10^ ^]^ as occurs with other MOF types, for instance, amino groups.^[^ ^11^ ^]^ Thus, it has demonstrated potential as an adsorbent for CO_2_ and as a membrane material for CO_2_ capture. Etxeberria‐Benavides et al.^[^ ^12^ ^]^ reported a three times higher CO_2_ permeability (from 770 to 2310 Barrer) when incorporating 40 wt.% of ZIF‐94 into 6FDA‐DAM as compared to the pure polymer. Pebax 1657 has also been employed as polymer matrix to embed ZIF‐94 particles, leading to a CO_2_/N_2_ selectivity of 36 (71% increment compared to the bare membrane) and a CO_2_ permeability of 137 Barrer (80% improvement) with a 10 wt.% loading.^[^ ^13^ ^]^ However, challenges persist in the practical implementation of MOFs, especially in MMMs and thin‐film nanocomposite membranes, due to issues such as inadequate compatibility with the polymer matrix, which can compromise membrane performance.^[^ ^14^ ^]^ Several research studies indicate that a close structural alignment between the polymer and fillers can be beneficial in establishing a compatible interfacial structure and enhancing the resistance to plasticization.^[^ ^15^, ^16^ ^]^

**Figure 1:**
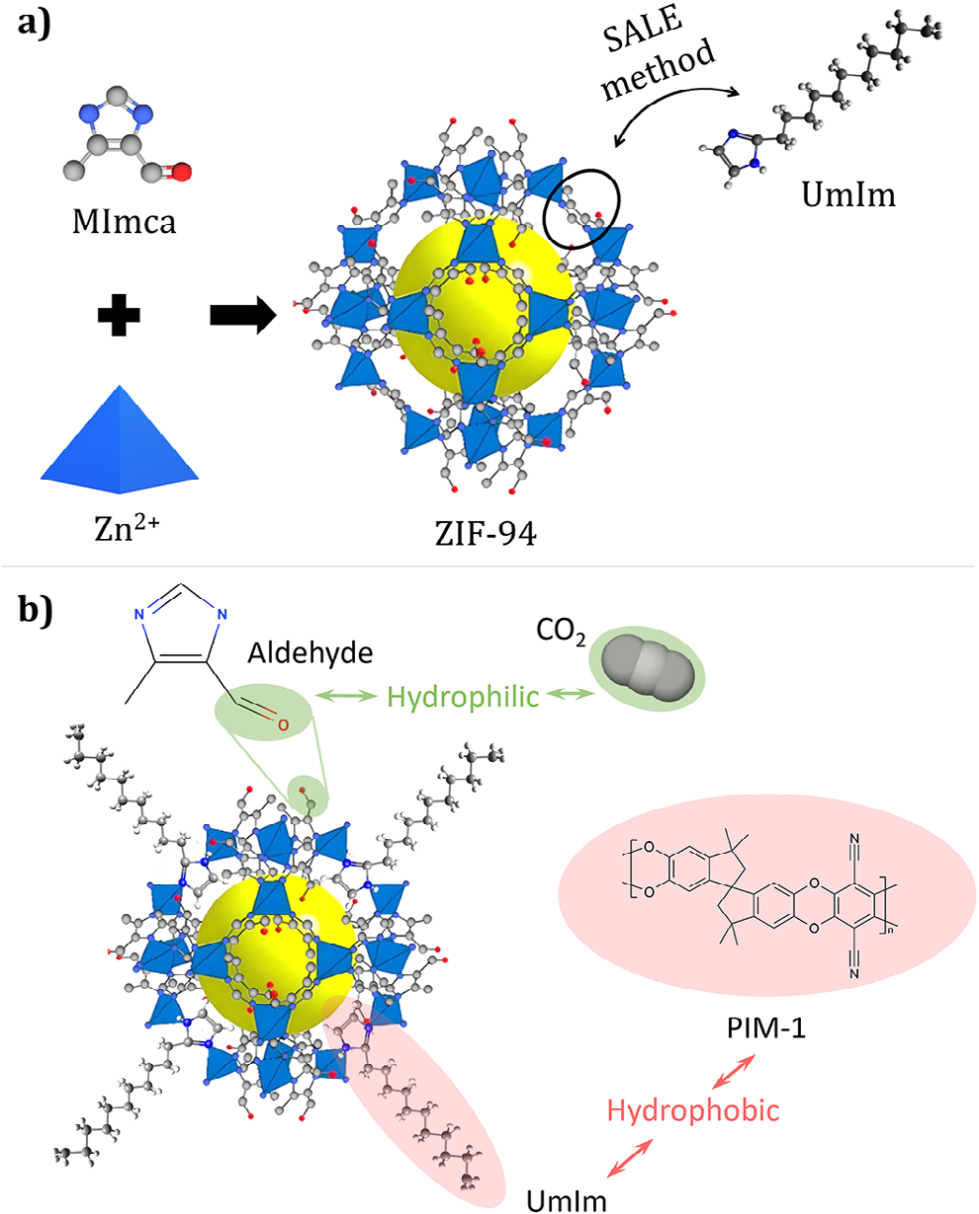
a) Scheme of the synthesis of ZIF‐94 and the subsequent solvent‐assisted ligand exchange (SALE) process with 2‐undecylimidazole and b) ZIF‐94‐umIm and its amphiphilic character: Hydrophobic part (due to the long hydrocarbon chain in 2‐undecylimidazole) colored in pink and hydrophilic components (due to the aldehyde group from ligand 4‐methyl‐5‐imidazolecarboxaldehyde in ZIF‐94) in green.

In this study, inspired by the success previously achieved with 2‐undecylimidazole (umIm)‐modified ZIF‐8/PIM‐1 MMMs,^[^ ^17^ ^]^ the SALE technique has been leveraged to produce an amphiphilic MOF by partially modifying the CO_2_‐philic ZIF‐94. This modification enhances its compatibility with PIM‐1, while simultaneously preserving some of its initial functionalities (as shown in Figure 1a,b). Thus, high membrane performance is achieved through the optimization of both the intrinsic properties of the polymeric material and those of the nanofiller. A branched PIM‐1 sample topology has been specifically designed to enhance selectivity and reduce physical aging of the polymer matrix.^[^ ^18^, ^19^ ^]^ Taking advantage of the inherent CO_2_‐affinity of ZIF‐94, a modification with umIm was carried out to improve its external hydrophobic character and enhance its interaction with the polymer matrix. The modified ZIF‐94 (ZIF‐94‐umIm) can be considered as an amphiphilic MOF^[^ ^20^, ^21^ ^]^ with both hydrophilic (derived from the presence of an aldehyde group in the 4‐methyl‐5‐imidazolecarboxaldehyde ligand) and hydrophobic (due the long hydrocarbon chain in 2‐undecylimidazole) organic moieties. It is noteworthy that this work reports the first successful synthesis of ZIF‐94‐umIm, which, to the best of our knowledge, cannot be achieved through standard nucleation and growth methods, as well as the first (PIM‐1)‐based MMM including an amphiphilic MOF. This study presents a comparative analysis between MMMs containing the original ZIF‐94 and the modified version, aiming to elucidate the impact of the modification on filler‐polymer compatibility and gas separation membrane performance.

## Results and Discussion

### Synthesis of Branched PIM‐1

The polymer synthesis procedure employed to obtain a branched PIM‐1 polymer, B‐PIM‐1, is outlined in Section 1.1 in Supporting Information (SI). Modifications which produced branched PIM‐1 samples have been previously reported in the literature,^[^ ^18^, ^19^, ^22^, ^23^ ^]^ with the underpinning theory to produce highly branched PIM‐1, outlined in a more recent publication.^[^ ^24^ ^]^ The polymer was purified and then characterized as described in Sections 1.1.3 and 1.1.4 in the Supporting Information. Size exclusion chromatography (SEC) analysis determined the weight‐average molar mass (*M*_w_) of polymer at 69 900 g mol^−1^ and dispersity (*Đ*) at 2.1; Lorentz peak fitting of aromatic proton NMR region of the polymer determined the level of branching at 10.9%; the amount of colloidal network content determined by filtration was 10%.

### MOF Characterization

**Figure 2:**
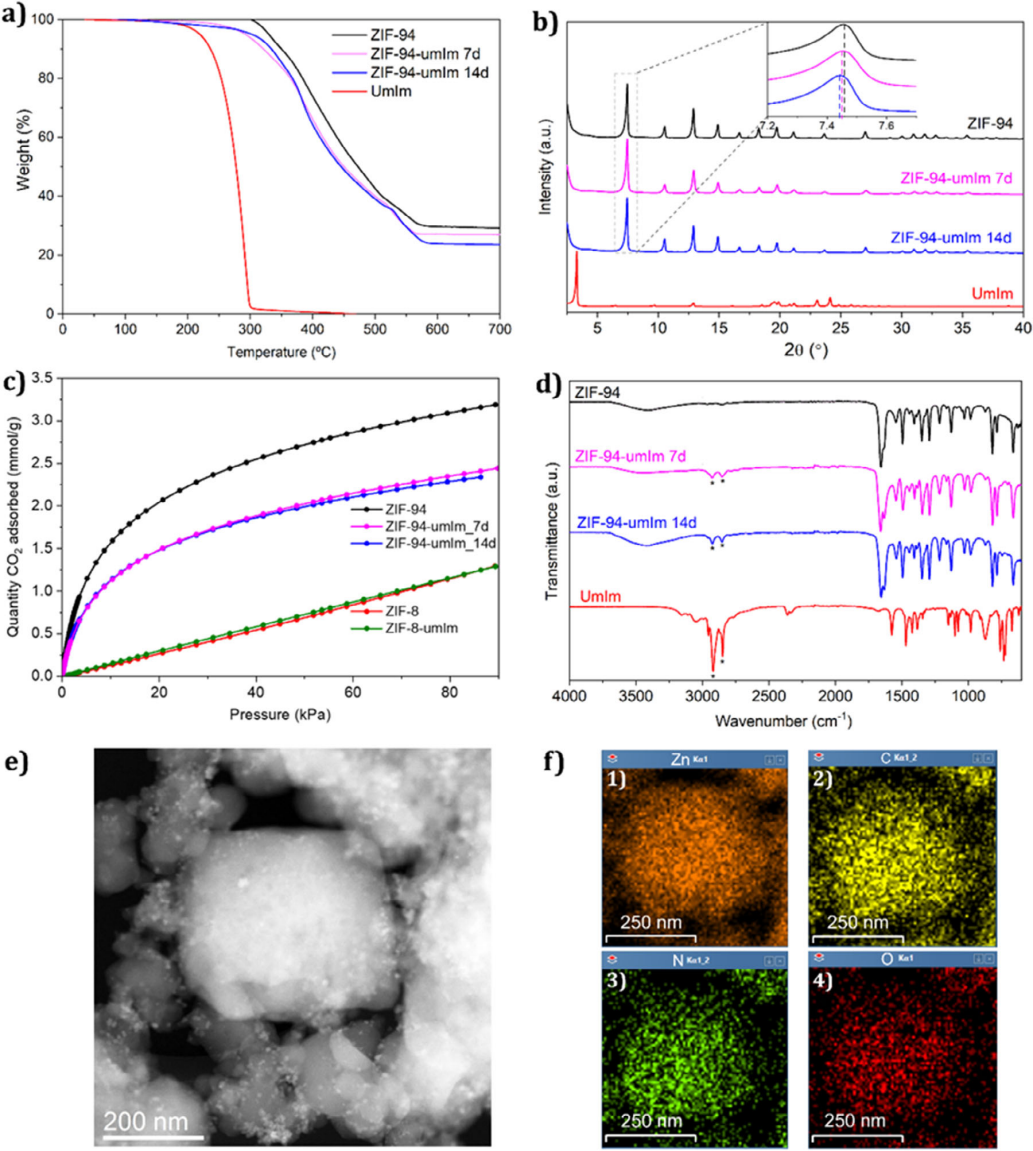
a) TGA data, b) XRD patterns, c) CO_2_ adsorption isotherms of ZIF‐94 and ZIF‐8 before and after SALE treatment with umIm, and d) FTIR spectra of ZIF‐94 (black), ZIF‐94‐umIm (7d) (pink), and ZIF‐94‐umIm (14d) (blue). UmIm (red) was also included in panels a, b, and d for comparison. e) TEM image of ZIF‐94‐umIm (14d) used for EDS measurement and f) EDS analysis of Zn atoms in orange (1), C atoms in yellow (2), N atoms in green (3), and O atoms in red (4).

ZIF‐94 particles previously synthesized were used for the SALE method (Section 1.2 and 1.3 in Supporting Information) and were also characterized for comparison with the modified sample. Two modified ZIF‐94 samples, obtained at different exchange times, were characterized and designated as ZIF‐94‐umIm (7d) and ZIF‐94 (14d), corresponding to exchange durations of 7 and 14 days, respectively. TGA data in Figure 2a show a decrease in thermal stability of the modified ZIF‐94 compared to the original ZIF‐94. The decrease in the degradation temperature may be attributed to the presence of the ligand umIm, which slightly reduces the stability of the well‐defined structure of ZIF‐94 by inducing some distortions in the ZIF framework, while essentially preserving the original crystal structure of ZIF‐94. TGA curves do not suggest the encapsulation of the ligand (e.g., with a weight loss step occurring at a temperature slightly above that corresponding to the pure ligand), which is consistent with a structural modification of the MOF. The plot corresponding to the derivative of the mass loss versus temperature (Figure S4) shows a sharp decomposition peak at 290 °C for pure umIm. If free ligand were present in ZIF‐94‐umIm, a similar peak would appear; however, no such peak is observed in the corresponding spectra. Instead, both ZIF‐94 and ZIF‐94‐umIm spectra indicate that umIm decomposes alongside the original mImca ligand, which is coordinated to Zn^2+^. This stabilization at similar temperatures suggests that umIm is also coordinated to Zn^2+^. The step at above 50 °C of around 10 wt.% is typically observed for this particular MOF as its hydrophilic nature results in water retention.^[^ ^25^ ^]^ The weight value at 700 °C, attributed to ZnO as a result of the degradation of ZIF under air, is lower for ZIF‐94‐umIm as compared to ZIF‐94. In other words, the organic part in ZIF‐94‐umIm increased compared to ZIF‐94, confirming the substitution of umIm. These weight loss values (previously normalized at 150 °C to discard the amount of solvent) were also used to estimate the percentage of substitution. The theoretical amount of ZnO present after thermal degradation due to the TGA experiment generated in air was calculated for ZIF‐94, Zn(mImca)_2_ (0% ligand exchange), and Zn(umIm)_2_ (for a hypothetical 100% ligand exchange). Considering 100 g of Zn(mImca)_2_ and Zn(umIm)_2_, the generated ZnO that would be obtained in TGA in air would be 28.7 and 16.1 g, respectively. The measured values in the TGA analyses for the ZIF‐94‐umIm (7d) and ZIF‐94‐umIm (14d) samples were 23.8 and 27.0 g ZnO, respectively. Interpolating these results indicate that the ligand exchange achieved was 18% and 39% for ZIF‐94‐umIm (7d) and ZIF‐94‐umIm (14d), respectively.

The XRD pattern in Figure 2b reveals that ZIF‐94‐umIm maintained the crystallinity of the original ZIF‐94 after the ligand exchange, with its main peaks at 7.45°, 10.5°, and 12.9°. However, as shown in the inset of Figure 2b, a slight shift was observed from 7.46° (ZIF‐94) to 7.45° (ZIF‐94‐umIm (7d)) and further to 7.44° (ZIF‐94‐umIm (14d)). This corresponds to a slight increase in the unit cell parameter, consistent with the integration of the bulkier ligand umIm and, consequently, with an expansion of the cell volume.

N_2_ adsorption isotherms were obtained to calculate the BET specific surface area (SSA) values. These indicate a reduction in this parameter after the SALE treatment, decreasing from 366 m^2^ · g^−1^ for the original ZIF‐94 to 285 and 274 m^2^ · g^−1^ for ZIF‐94‐umIm at 7 days and 14 days, respectively. This decrease is attributed to a blockage of the pore window caused by the aliphatic chain present in umIm, coherent with a successful ligand exchange in the outer region of the ZIF crystals. CO_2_ adsorption isotherms, shown in Figure 2c, were also acquired. Although SSA may be relatively low, CO_2_ adsorption revealed considerably high values at 90 kPa and 0 °C: ZIF‐94 adsorbed 3.19 mmol · g^−1^, which decreased after SALE treatment with umIm to 2.34 mmol · g^−1^ (Table 1). The replacement of the aldehyde group in ZIF‐94 with the hydrophobic alkyl chain of the umIm ligand leads to a reduction in CO_2_ sorption, as expected. However, a mere encapsulation of umIm in the ZIF (i.e., umIm solely in the cavity of ZIF‐94 and not exchanged) structure would have produced a much important reduction of the BET SSA and adsorption capacity of ZIF‐94‐umIm. In summary, these CO_2_ sorption values demonstrated that even after the SALE treatment, both ZIF‐94‐umIm (7d and 14d) samples seem to be suitable materials for CO_2_ capture. In fact, despite having a much lower SSA than, for example, ZIF‐8 and its modified version ZIF‐8‐umIm, ZIF‐94‐umIm shows higher CO_2_ adsorption values (collected in Table 1). CO_2_ adsorption experiments (Figure S5) were also employed to assess the pore size using the Horvath–Kawazoe (HK) model, as described elsewhere.^[^ ^26^, ^27^ ^]^ The pore sizes of both ZIF‐94‐umIm (7d) and ZIF‐94‐umIm (14d) show a slight shift toward larger pore widths compared to ZIF‐94. This trend aligns with the XRD analysis, which suggests a very small cell volume expansions and therefore slightly wider pores.

**Table 1:** CO_2_ adsorption at 0 °C and 90 kPa and BET specific surface area of ZIF‐94 and ZIF‐8 before and after SALE treatment with umIm.

Sample	CO_2_ adsorption (mmol · g^−1^)	BET SSA (m^2^ · g^−1^)
ZIF‐94	3.19	366
ZIF‐94‐umIm (7d)	2.44	285
ZIF‐94‐umIm (14d)	2.34	274
ZIF‐8	1.30	1444
ZIF‐8‐umIm	1.29	573

Figure 2d includes the FTIR spectra of ZIF‐94, ZIF‐94‐umIm (7d and 14d) and pure umIm. Both ZIF‐94‐umIm (7d and 14d) reveal the presence of peaks at 2850 and 2900 cm^−1^ (indicated with asterisks in the figure), corresponding to C─H bonds characteristics of the aliphatic chains from umIm. This further confirms the exchange with umIm. The remaining peaks are consistent with those typical of ZIF‐94.

High resolution‐transmission electron microscopy (HR‐TEM) images were used to elucidate the shape and composition of the ZIF‐94‐umIm (14d) nanoparticles. Figure 2e shows a nanoparticle of around 300 nm, which was employed for energy dispersive X‐ray spectroscopy (EDS) analysis, shown in Figure 2f. The lower density of oxygen atoms (red dots in Figure 2f) in the external part of the nanoparticle suggests that the ligand exchange primarily occurs in the outer side of the nanoparticle. This observation is consistent with the replacement of the original ZIF‐94 (mImca ligand), which contains an aldehyde group, by the exchanged ligand (umIm), which lacks oxygen atoms. Since the external surface of ZIF‐94 particles is the most exposed, the ligand exchange should mostly take place there. Additionally, the introduced linker may be quite large to easily pass through the narrow pore windows in ZIF‐94.^[^ ^17^, ^28^ ^]^ TEM images also revealed tiny nanoparticles of around 5 nm surrounding the MOF nanoparticles (clearly visualized in Figure S6). According to the EDS composition, these nanoparticles may be related to Zn(OH)_2_ nanoparticles and the possible formation due to the addition of NaOH during the ZIF‐94 synthesis step. SEM images did not show them because of lower resolution and magnification, yet the resolution of TEM allowed their clear visualization.

SEM images in Figure S7 show that ZIF‐94 (Figure S7a) did not suffer significant morphological changes after SALE treatment (Figure S7b). Both are around 300 nm in particle size, with the typical rhombododecahedral shape of ZIF‐94.^[^ ^29^ ^]^ These results confirm the reported observations of maintaining the same morphology and particle size during ligand exchange.^[^ ^30^ ^]^

Dynamic light scattering measurements were also performed to obtain a broad‐scale perspective on particle size distribution of both original ZIF‐94 and its modified version. Figure S8 shows a similar particle size distribution of both samples, with the highest particle population observed at around 400 nm.

Elemental analysis was conducted for sample ZIF‐94‐umIm (14d) to quantify the changes in carbon content after the SALE treatment, which concerns the ligand exchange between mImca and umIm. Table S1 collects the wt.% of C, H, and N in the sample after SALE treatment (measured by elemental analysis) and theoretical amounts in ZIF‐94 (Zn(mImca)_2_, 0% exchange) and in a hypothetical ZIF made entirely of umIm, representing 100% of exchange (Zn(umIm)_2_). The exchange percentage was calculated using the theoretical wt.% of ZIF‐94 (0% exchange) and ZIF‐94‐umIm (100% exchange) and interpolating the carbon wt.% experimentally obtained for the SALE ZIF‐94‐umIm, as shown in the Supporting Information (Table S1 and Equations S1, S2). This calculation reveals that ZIF‐94‐umIm (14d) contains roughly 30 wt.% of umIm ligand substitution, which is consistent with the findings obtained through TGA analysis.

The amphiphilic behavior of the modified MOF was evaluated by its interaction with different solvents, which is shown in Figure S9.

### MMM Characterization

**Figure 3:**
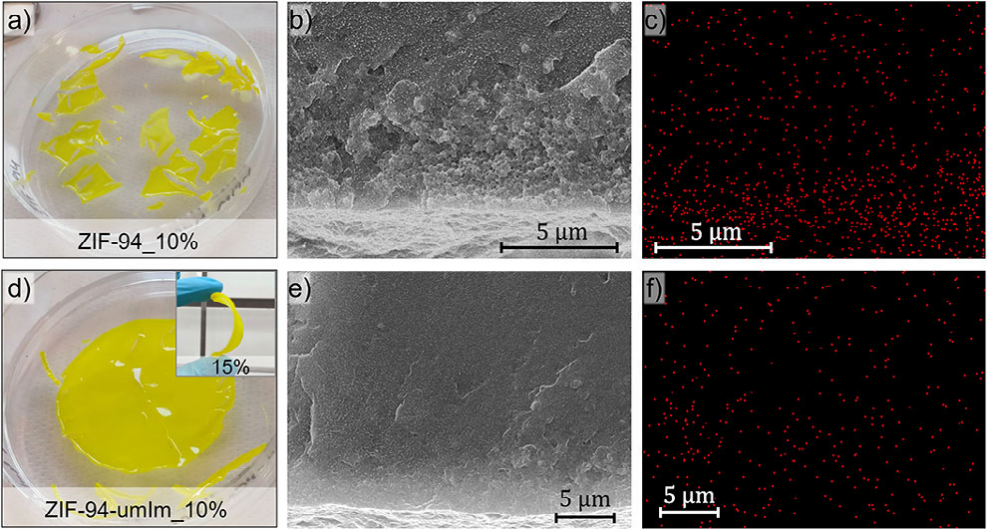
Pictures of membranes with different fillers and loadings: a) 10 wt.% of ZIF‐94 and (d), 10 wt.% of ZIF‐94‐umIm (inset: membrane with15 wt.% ZIF‐94‐umIm). SEM images of: b) PIM‐1 with ZIF‐94 (10 wt.%) and e) PIM‐1 with ZIF‐94‐umIm (10 wt.%), along with their corresponding EDS mapping analysis: c) PIM‐1 with 10 wt.% ZIF‐94 and f) PIM‐1 with 10 wt.% ZIF‐94‐umIm.

The fully characterized ZIF‐94‐umIm (14d) was evaluated as a filler to produce MMMs with PIM‐1 (Section 1.4 in Supporting Information). From now on, ZIF‐94‐umIm (14d) will be referred to simply as ZIF‐94‐umIm, as MMMs were only prepared with this sample and not with ZIF‐94‐umIm (7d). Both ZIF‐94‐umIm (7d and 14d) exhibit comparable CO_2_ sorption capabilities, but the 14‐day sample features a greater extent of ligand exchange, making it more suitable for MMM fabrication. In this work, B‐PIM‐1 was used for preparing MMMs, but from now on, it will be named as PIM‐1 for simplicity. ZIF‐94 was also employed as a filler for comparative analysis. Membranes with 10 wt.% loading of ZIFs were characterized to understand the significant changes in the influence of each filler. At first glance, the consistency of MMMs with both fillers is significantly different. Figure 3a suggests poor filler‐polymer compatibility for a membrane with ZIF‐94 at 10 wt.%, resulting in increased fragility and susceptibility to rupture, hindering its gas separation testing. In contrast, MMMs containing ZIF‐94‐umIm demonstrated superior mechanical stability and flexibility compared to ZIF‐94 MMMs at the same loading (Figure 3d) and even at higher loadings (inset in Figure 3d, with a loading of 15 wt.%).

SEM was performed together with EDS mapping to analyze the presence of the filler within the membrane. Figure 3b,e show the SEM images used to measure the EDS. Both correspond to cross‐sectional images of membranes with 10 wt.% loading of ZIF‐94 and ZIF‐94‐umIm, respectively. Figure 3c,f depict the Zn atoms from EDS measurements as red dots. Figure 3c shows more density of dots in the lower part of the membrane with ZIF‐94, which suggests that during the membrane formation the nanofillers agglomerate and sediment at the bottom of the Petri dish. Agglomeration occurs due to poor compatibility with the polymer chain (i.e., stronger interactions between ZIF‐94 nanoparticles compared to ZIF‐94/PIM‐1). On the contrary, the membrane with ZIF‐94‐umIm as a filler, suggests a better dispersion of the nanoparticles as shown in Figure 3f. This is attributed to the higher compatibility between the hydrophobic modification of the filler and the polymer chains, which led to a better stability of the dispersion of the modified MOF during membrane formation.

**Figure 4:**
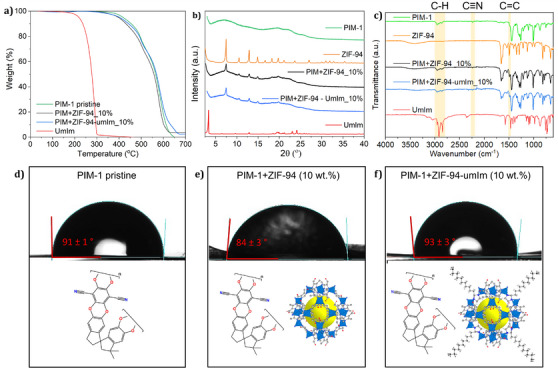
a) TGA curves, b) XRD patterns, and c) FTIR spectra of pristine PIM‐1 (green line), PIM‐1 with 10 wt.% of ZIF‐94 (black line), PIM‐1 with 10 wt.% of ZIF‐94‐umIm (blue line), and undecylimidazole (red line). Contact angle images of a water drop of 4 µL in PIM‐1 membranes: d) Without filler, e) with 10 wt.% of ZIF‐94, and f) with 10 wt.% of ZIF‐94‐umIm.

Compared with pristine PIM‐1 membrane, TGA curves in Figure 4a reveal a slightly lower stability when the PIM‐1 membranes incorporate ZIF‐94 and a slightly higher with ZIF‐94‐umIm. When focusing on the derivative curves of TGA performed in membranes (Figure S10), pristine PIM‐1 reveals a degradation temperature of 585 °C. When these membranes include ZIF‐94 as a filler, this temperature slightly decreases to 579 °C, and including ZIF‐94‐umIm as filler, the temperature slightly increases up to 586 °C. This can be related to the compatibility between PIM‐1 and each filler: Worse compatibility between polymer and ZIF‐94 leads to reduced thermal stability of polymer backbone, whereas a better ZIF‐94‐umIm/PIM‐1 compatibility results in higher energy required to degrade polymer chains. The residual values observed at 700 °C of MMMs with ZIF‐94 and ZIF‐94‐umIm were 3.7% and 2.5%, respectively. These amounts represent the ZnO produced from the TGA experiment under air. Considering 10 wt.% of ZIF loading in both samples, the expected amounts of ZnO would be 2.9% for ZIF‐94 and 2.2% for ZIF‐94‐umIm (assuming a 39% of substitution and thus, a molar weight of 370.58 g mol^−1^). These theoretical results are pretty similar to the ones obtained by TGA, confirming the MMM loadings. XRD spectra (Figure 4b) exhibit the main peaks of ZIF‐94 and ZIF‐94‐umIm in both MMMs with 10 wt.% of ZIF loading, proving the presence of crystalline nanoparticles within the polymer matrix. FTIR spectra in Figure 4c reveal the characteristics peaks reported in literature for PIM‐1 membranes: the aliphatic and aromatic C─H stretching (around 2800– 3010 cm^−1^), nitrile groups (C≡N, at around 2240 cm^−1^) and C═C aromatic bending (at around 1600 cm^−1^).^[^ ^31^ ^]^ Besides, MMMs containing ZIF‐94 and ZIF‐94‐umIm also reveal peaks at 1660 cm^−1^ corresponding to C═O present in the ZIFs.

Water contact angle was measured to understand the influence on MMM hydrophobicity when incorporating the original ZIF‐94 compared to the modified one (ZIF‐94‐umIm). The contact angle value was obtained as the average of four measurements in different areas of the membrane. Pristine PIM‐1 membrane revealed a contact angle of 91 (±1)° (Figure 4d), while MMMs including ZIF‐94 (10 wt.%) showed 84 (±3)° (Figure 4e) and MMMs with ZIF‐94‐umIm (also 10 wt.%) exhibited 93 (±3)° (Figure 4f). Even though the filler loading was relatively low (10 wt.%), it already showed a slight influence on the membrane hydrophobicity. These values are not very far from those obtained for MMMs with ZIF‐8 (92°) and ZIF‐8‐umIm (97°),^[^ ^17^ ^]^ confirming that the molecule (2‐undecylimidazole) used for the ZIF hydrophobization produces analogous effects on the filler‐polymer interfaces for both ZIF MMMs (ZIF‐94‐umIm and ZIF‐8‐umIm).

The viscosity of the casting solutions was measured to gain insight into the interactions between the polymer and different fillers. Viscosity tests (Figure S11) were performed in casting solutions of pristine PIM‐1, PIM‐1 with ZIF‐94 (10 wt.%), and PIM‐1 with ZIF‐94‐umIm (10 wt.%). Pristine PIM‐1 shows a viscosity value of 1.84 cP (mPa·s), while the addition of nanofillers increases this value up to 1.94 cP for ZIF‐94‐umIm and up to 2.16 cP for ZIF‐94. This suggests that the addition of nanofillers hinders the movement of polymer chains. A lower viscosity value when adding ZIF‐94‐umIm, as compared to ZIF‐94, could be attributed to a better interaction with PIM‐1, which is translated into a better homogeneity of the membranes with this ZIF. Prior researches have demonstrated that strong interactions between polymers and filler nanoparticles lead to a decrease in the viscosity of the casting solutions.^[^ ^32^, ^33^ ^]^

Tensile traction tests were performed to study the mechanical properties of the membranes. The addition of the nanofiller slightly enhanced the Young's modulus, increasing from 273 (± 50) MPa for pure PIM‐1 to 286 (± 63) MPa and 382 (± 75) MPa for MMMs containing ZIF‐94 and ZIF‐94‐umIm, respectively.

### Gas Separation

**Figure 5:**
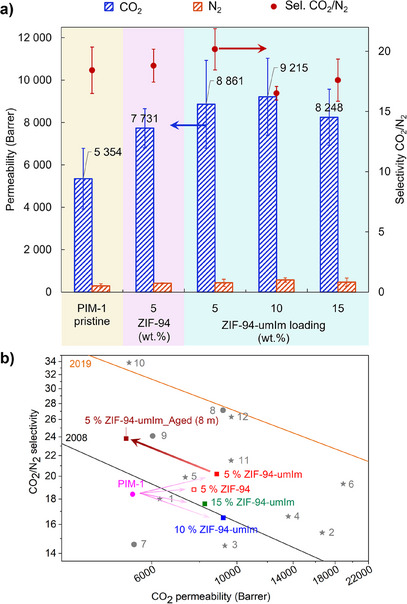
a) Gas separation performance of PIM‐1 membranes for 15/85 CO_2_/N_2_ mixed gas separation as a function of filler: Without filler (yellow background), with ZIF‐94 at 5 wt.% loading (pink background) and with different ZIF‐94‐umIm loading (blue background). b) Robeson upper bound^[^ ^6^, ^34^ ^]^ from 2008 (grey line) and from 2019 (orange line) for CO_2_/N_2_ separation and comparison with MMMs prepared in this work for both original ZIF‐94 (5 wt.%) and ZIF‐94‐umIm (5, 10, and 15 wt.%, including the 8‐month aging MMM shown in Table 2) and selected data from bibliography. Stars 1–6: PIM‐1 with ZIF‐8;^[^ ^35^ ^]^ PIM‐1 with ZIF‐8‐umIm;^[^ ^17^ ^]^ PIM‐1, 6FDA‐DAM, and ZIF‐8;^[^ ^36^ ^]^ PIM‐1;^[^ ^37^ ^]^ PIM‐PI‐EA,^[^ ^38^ ^]^ and CPIM‐ 1,^[^ ^39^ ^]^ respectively), and stars 10–12: PIM‐1 with 8% UiO‐66‐(CF_3_)_2,_^[^ ^40^ ^]^ PIM‐1 with polyUiO‐66(1:4),^[^ ^41^ ^]^ and PIM‐1 with 6% TpTta‐COF^[^ ^42^ ^]^). Novel polymer materials are represented by dots 7–9: CANAL‐Me‐Me_2_F,^[^ ^43^ ^]^ PIM‐BTrip,^[^ ^45^ ^]^ and TTA‐100.^[^ ^44^ ^]^

Gas separation measurements were performed as explained in Section 1.7 in Supporting Information. A comparison between MMMs containing both fillers, ZIF‐94 and ZIF‐94‐umIm, is shown in Figure 5a. Mixed gas experiments using CO_2_/N_2_ (15/85), the composition being typical of CO_2_ capture applications, were conducted at 35 °C. The comparative analysis between both types of MMMs had to be done at 5 wt.% loading since membranes containing ZIF‐94 at higher loadings were extremely fragile and broke upon testing, as explained above (Figure 3). The gas separation performance of PIM‐1 is highly dependent on the synthetic conditions employed. In this particular case, PIM‐1 has been optimized through the manipulation of the polymer microstructure to exhibit competitive performance.^[^ ^18^, ^19^ ^]^ The pristine PIM‐1 membranes show a CO_2_ permeability of 5300 Barrer and a CO_2_/N_2_ selectivity of 18.4. MMMs having 5 wt.% of ZIF‐94 exhibit an increase in the permeability of CO_2_ to 7700 Barrer, while maintaining the CO_2_/N_2_ selectivity at 18.8. When incorporating ZIF‐94‐umIm into PIM‐1 at the same loading (5 wt.%), the permeability of CO_2_ increases (8900 Barrer) as compared to the original PIM‐1 and ZIF‐94 MMMs. The CO_2_/N_2_ selectivity was also improved, achieving a value of 20.2. These results confirm an enhancement of the gas separation performance; i.e., 68% higher permeability of CO_2_ and 10% higher CO_2_/N_2_ selectivity than the pristine PIM‐1 membrane. This is attributed to the presence of a CO_2_‐philic porous nanofiller, which has an appreciable effect even at a low loading. As compared to the original ZIF‐94, the modified ZIF‐94 allows for a better dispersion within the polymer matrix, thereby reducing the likelihood of agglomeration and the potential creation of nonselective voids. Consequently, ZIF‐94‐umIm nanofillers facilitate the formation of faster gas molecule pathways than ZIF‐94. When a 10 wt.% loading of ZIF‐94‐umIm is used, CO_2_ permeability increases above 9200 Barrer with a slight decrease in CO_2_/N_2_ selectivity down to 16.5. As the nanofiller loading increases, the likelihood of agglomeration also rises, which can lead to the formation of unselective interfacial voids and inability to fully utilize the properties of the nanofiller. MMMs with 15 wt.% loading show a decrease in CO_2_ permeability compared to those with 5–10 wt.% loading, down to 8250 Barrer, while the selectivity is maintained at similar values (17.6).

The performance of the membranes studied in this work are collected in Figure 5b along with the Robeson upper bound of 2008 and 2019. Both membranes with 5 wt.% loading (with ZIF‐94 and ZIF‐94‐umIm) overcome the Robeson upper bound of 2008, as both maintain the selectivity of PIM‐1 while increasing considerably the permeability of CO_2_. The CO_2_‐philic character of ZIF‐94 notably increase the permeability of this gas. ZIF‐94‐umIm, which decreases its CO_2_‐philic character due to the substitution of mImca ligand by umIm (as shown above in Table 1) provides in turn amphiphilic properties to the nanofiller essential for its enhanced processing when applied to MMMs. The gas separation performance of this MMMs demonstrate that improving filler dispersion and compatibility within the polymer matrix is worthwhile even at the expense of slightly sacrificing part of its CO_2_‐philic nature. Several studies are also included in Figure 5b as comparison (with permeability data collected in Table S2), most of them involving PIM‐1 or ZIF nanofillers. The results shown in this manuscript are seen in this figure to be among the best, with a good permeability‐selectivity balance. Adding the nanofiller to PIM‐1 membranes significantly improves its performance, approaching the 2019 upper bound defined by advanced polymers such as B‐Trip (dot 8 in Figure 5b).^[^ ^45^ ^]^

Selected membranes (PIM‐1 and MMM with a 5 wt.% loading) were measured after 8 months of physical aging. CO_2_ permeabilities and CO_2_/N_2_ selectivities were measured as collected in Table 2. Pristine PIM‐1 membrane decreased its CO_2_ permeability by 45%. MMM with ZIF‐94‐umIm decreases its CO_2_ permeability in a similar value (49%), while the MMM with ZIF‐94 suffered a slightly higher decrease of around 60%. The addition of nanofillers introduces additional free volume which leads to higher initial permeabilities in comparison with pristine PIM‐1. Physical aging consists of a reorganization of polymer chains, driven by excess free volume. Thus, this phenomenon becomes more pronounced in membranes with a higher free volume, which provides an explanation for the higher CO_2_ permeability losses observed in MMMs. However, nanofillers can also hinder the polymer chain reorganization through their interactions with the polymer chains.^[^ ^46^, ^47^ ^]^ When comparing both MMMs, ZIF‐94‐umIm exhibits a lower reduction in CO_2_ as compared to ZIF‐94. This finding aligns with the hypothesis that ZIF‐94‐umIm displays enhanced compatibility with the polymer, thereby impeding the movement of polymer chains more efficiently. CO_2_/N_2_ selectivities increased by 13% and 14% in pristine PIM‐1 membrane and MMMs including ZIF‐94‐umIm, respectively, while with the MMMs with ZIF‐94 increased by 20%. This makes the MMMs with 5% of ZIF‐94‐umIm to surpass the Robeson upper bound of 2008 after 8 months of physical aging (Figure 5b), despite their decrease in CO_2_ permeability over time.

**Table 2:** CO_2_ permeabilities and CO_2_/N_2_ selectivities measured (at 35 °C for a 15/85 CO_2_/N_2_ feed mixture) in fresh membranes and after 8 months from their formation.

Membrane	Date	CO_2_ permeability (Barrer)	CO_2_/N_2_ selectivity
Pristine PIM‐1	Fresh	4020	19.9
	After 8 months	2200 (↓ 45%)	22.5 (↑ 13%)
PIM‐1 + 5 wt.% ZIF‐94	Fresh	6700	19.2
	After 8 months	2680 (↓ 60%)	23.1 (↑ 20%)
PIM‐1 + 5 wt.% ZIF‐94‐umIm	Fresh	10 060	20.8
	After 8 months	5150 (↓ 49%)	23.8 (↑ 14%)

The performance of MMMs can substantially vary for different PIM‐1 samples due to distinct polymer microstructures, which are highly sensitive to reaction conditions.^[^ ^19^ ^]^ The polymerization conditions employed in this case enabled the production of a branched PIM‐1 polymer,^[^ ^24^ ^]^ which has been shown to age more slowly and show better miscibility with fillers in membranes.^[^ ^48^ ^]^ In order to facilitate a comparison of the performance of different MMMs, a “score improvement” index is frequently employed with the aim of standardizing the results.^[^ ^49^ ^]^ This index represents the perpendicular distance between a data point and the upper bound limit. Figure S12 illustrates the score improvement for several MMMs composed of PIM‐1 and various ZIF nanoparticles, including ZIF‐8, ZIF‐67, ZIF‐71, ZIF‐94, and functionalized derivatives of these ZIFs. In contrast to other glassy polymers, where the addition of porous nanofillers has been shown to significantly enhance membrane permeability due to increased transport rates, MMMs containing PIMs necessitate an alternative approach. The high gas diffusivity observed in PIM‐1 is attributed to its large excess free volume, which makes it challenging to further enhance the permeability of the material by adding porous nanofillers. Nanofillers that are CO_2_‐philic, such as those that have been functionalized with hydrophilic moieties^[^ ^50^ ^]^ or that contain unsaturated metal centers,^[^ ^51^ ^]^ are highly advantageous. However, the rigid structure of PIM‐1 is susceptible to the formation of interfacial defects due to the inadequate polymer‐filler interactions. These defects typically lead to increased permeability but at the expense of selectivity. This decline in selectivity is especially problematic for PIM‐1, as it already demonstrates high permeability but exhibits moderate selectivity. Consequently, the score index frequently demonstrates minimal or, in some instances, even negative improvement. This behavior is not observed in MMMs incorporating ZIF‐94‐umIm up to loadings of 10 wt.%. This provides an explanation for the findings that PIM‐1/ZIF‐94‐umIm MMMs demonstrate one of the highest score index in Figure S12. ZIF‐94‐umIm nanoparticles exhibit amphiphilic behavior, forming stable dispersions in chloroform despite the presence of hydrophilic moieties and exhibiting particularly high CO_2_ permeation properties. This demonstrates the versatility of the ligand exchange technique to manipulate the MOF properties.

Pure PIM‐1 and MMMs in this study were also tested with CO_2_/CH_4_ (50/50, v/v) mixtures at one specific loading (5 wt.%) to compare their performance with different gas mixtures. Figure S13 shows gas permeabilities with their corresponding CO_2_/N_2_ and CO_2_/CH_4_ selectivities. The CO_2_ permeability was similar in both cases, while the selectivity decreased (from 18.0 of CO_2_/N_2_ selectivity to 12.8 of CO_2_/CH_4_) when CH_4_ competed with CO_2_ for sorption sites within the polymer matrix. Thus, the higher CH_4_ permeability, as compared to N_2_, can be associated with the higher solubility of this gas in PIM‐1.^[^ ^52^ ^]^

Gas transport in PIM‐1 is described by the solution‐diffusion model. Time‐lag measurements were used to investigate the enhanced CO_2_ permeation in MMMs containing ZIF‐94‐umIm. The findings suggest that incorporating the nanofiller significantly increases gas diffusivity compared to pure PIM‐1 (Table 3). ZIF‐94‐umIm exhibit strong affinity for CO_2_, allowing gas molecules to penetrate their pores. This facilitates enhanced transport due to the creation of molecular pathway shortcuts through the MOF pores.^[^ ^50^ ^]^ In MMMs, additional porosity is generated at the interface between the rigid particles of ZIF and the organic matrix (PIM‐1) due to disruption in polymer packing, offering reduced resistance to gas transport. However, if nanoparticle agglomeration occurs, the polymer chains are often unable to rearrange around the rigid particles, leaving large voids that result in nonselective diffusion. This may account for the observed decrease in selectivity for MMMs at higher filler loadings (e.g., 15 wt.%), as illustrated in Figure 5a. Despite their high CO_2_ adsorption properties, the incorporation of ZIF‐94 and ZIF‐94‐umIm has a minimal influence on the solubility coefficient (Table 3). This is attributed to the inherently strong CO_2_ sorption properties of pure PIM‐1^[^ ^53^ ^]^ which accounts for its high permeability compared to smaller gas molecules with higher diffusivity, such as H_2_. Therefore, the addition of porous CO_2_‐philic materials primarily impacts the gas diffusivity more than the solubility.

**Table 3:** CO_2_ diffusion and solubility coefficients measured at 35 °C for PIM‐1 and MMMs containing ZIF‐94‐umIm after 6 months of physical aging.

Membrane	Solubility cm^3^ (STP) · cm^−3^ · atm^−1^	Diffusivity (10^−7^ cm^2^ · s^−1^)
Pristine PIM‐1	0.67	5.20
PIM‐1 + 5 wt.% ZIF‐94‐umIm	0.61	7.96

**Figure 6:**
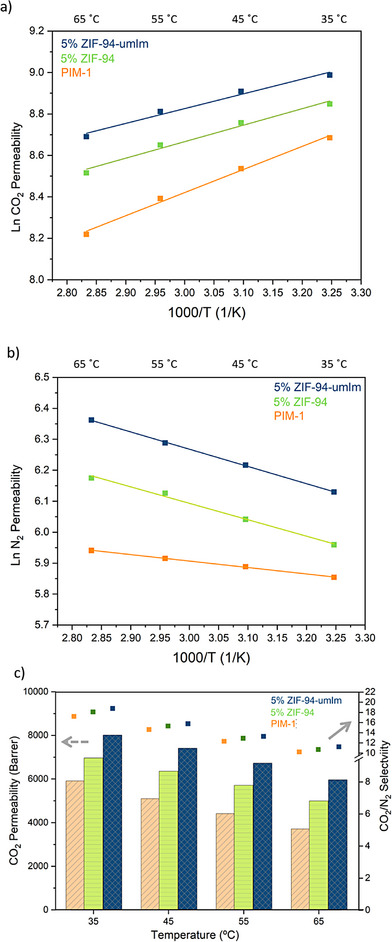
Logarithmic permeability of a) CO_2_ and b) N_2_ plotted against inverse temperature. c) Temperature‐dependent CO_2_ permeability and CO_2_/N_2_ selectivity of PIM‐1 and MMMs incorporating 5% ZIF‐94 and ZIF‐94‐umIm.

The effect of temperature on both MMMs and pure PIM‐1 was investigated for understanding temperature‐dependent properties such as sorption and diffusion. The diffusivity of all gases through a polymer membrane increases with temperature, as the higher thermal energy enhances the polymer chain mobility and reduces the diffusion hindrance for gas molecules. However, the sorption coefficient is influenced by the thermodynamic relationship between the permeant and the membrane material. The strong interaction between PIM‐1 and CO_2_ results in a negative sorption enthalpy,^[^ ^54^ ^]^ leading to an inverse relationship between temperature and the sorption coefficient i.e., as temperature increases, CO_2_ sorption decreases. In contrast, N_2_ sorption in PIM‐1 is barely affected. As a result, in PIM‐1 membranes (Figure 6a,b), the CO_2_ permeability decreases with increasing temperature, while the N_2_ permeability exhibits an upward trend. The permeability‐temperature dependence for all membranes follows the Arrhenius equation. Interestingly, both MMMs exhibit a similar trend, with their trajectories running parallel, as illustrated in Figure 6a,b. This suggests that ZIF‐94 and ZIF‐94‐umIm have a similar effect on the gas separation performance. Both MMMs increase CO_2_ and N_2_ permeabilities compared to the pure polymer. In other words, as the temperature increases, the CO_2_ permeability declines less while permeability of N_2_ rises more in MMMs than in pure PIM‐1. These findings suggest an enhanced porosity in MMMs as compared to pure PIM‐1. Moreover, this increased porosity exhibits a temperature‐activated transport, consistent with the gate‐opening effect of MOFs. The flexible linkers in ZIFs exhibit a phenomenon where molecules like N_2_ (3.64 Å), larger than the pore aperture (2.6 Å), can penetrate the pores due to their dynamic porosity. This effect is more pronounced at higher temperatures, as MOF linkers have increased energy to rotate. Figure 6c highlights the ability of the MOF to maintain an increased CO_2_ permeability compared to pure PIM‐1, minimizing the performance decrease at elevated temperatures.

Figure S14 illustrates the dependence of CO_2_ permeability on pressure. For all membranes, both CO_2_ permeability and CO_2_/N_2_ selectivity decrease, consistent with the behavior of glassy polymers. According to the dual‐mode sorption and diffusion model, increasing pressure leads to a significant reduction in Langmuir sorption sites, thereby lowering the solubility coefficient.^[^ ^53^ ^]^

**Figure 7:**
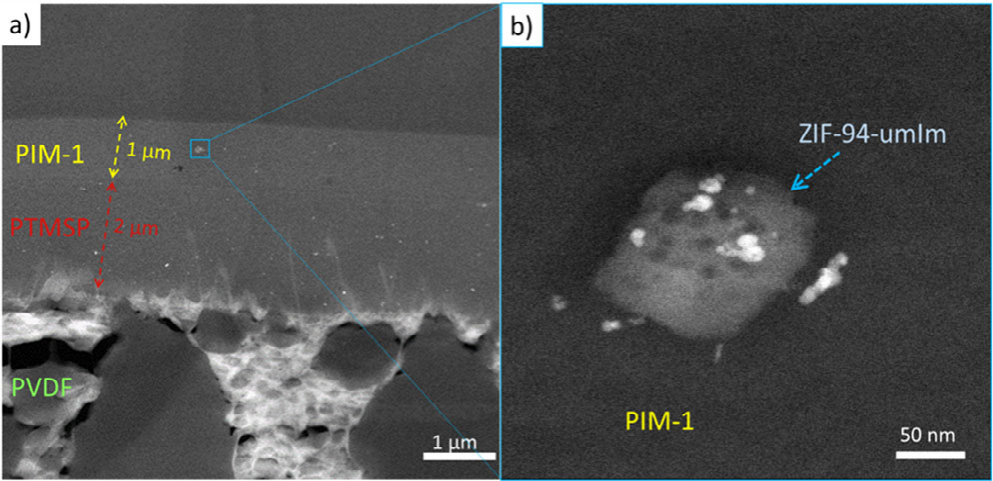
a) STEM‐High‐angle annular dark field (HAADF) images of a cross‐section of a TFN membrane and b) a detailed visualization of the ZIF‐94‐umIm within the polymer matrix. EDS analysis (Figure S17) confirms that the nanoparticle is primarily composed of Zn.

Thin‐film nanocomposite (TFN) membranes were fabricated to validate the feasibility of the proposed membrane fabrication approach with amphiphilic ZIF‐94‐umIm. The functionalization of the MOF facilitates the efficient integration of the nanofiller into the polymer matrix. Figure 7a shows the three‐layer structure of the TFN membrane, consisting of a polyvinylidene fluoride (PVDF) support with large pores, a poly(1‐trimethylsilyl‐1‐propyne) (PTMSP) gutter layer, and a PIM‐1 selective layer on the top. Energy dispersive X‐ray spectroscopy (EDS) analysis (Figure S15) confirms the presence of silicon in the gutter layer, aiding in the identification of the start and end of such layer. HR‐TEM images (Figure S16), which provided enhanced contrast compared to scanning transmission electron microscopy (STEM) images, were used to measure the thicknesses of the different layers, approximately 1 µm for the PIM‐1 layer and 2 µm for the PTMSP layer. The presence of ZIF‐94‐umIm nanoparticles within the polymer matrix can be depicted in Figure 7b. The resulting TFNs exhibit high CO_2_ permeance and satisfactory CO_2_/N_2_ selectivity, positioning them as promising candidates for CO_2_ capture applications (Figure S18). Initial measurements were performed using helium as the carrier gas to simplify gas chromatography operation. However, helium is rarely used in industrial applications and to better reflect real‐world conditions, TFNs were also tested without a carrier gas, as typically done in industrial processes. Additionally, TFNs were evaluated under varying pressures from 2 to 3.5 bar and with CO_2_/N_2_ compositions of 50:50 and 85:15. All TFN performances (1934–2350 GPUs and 20.0–23.5 selectivity) fall within the region of interest. However, the permeance of the PIM/ZIF‐94‐umIm layer appears lower than the theoretically expected value for a 1 µm thick layer, with a CO_2_ permeability of approximately 8000 Barrer. This observation is a common issue when preparing thin layers of microporous polymers, as previously studied by Lee et al.^[^ ^55^ ^]^ Additionally, MMMs in this study are soaked in methanol and subjected to vacuum treatment to increase the free volume and, consequently, permeance. In contrast, TFNs are measured without any treatment, in accordance with standard methodology used by most authors.

To highlight the amphiphilic nature of ZIF‐94‐uIm, MMMs were fabricated using PEBAX 3533, a hydrophilic polymer, as the base material (Figure S19). The incorporation of ZIF‐94 and ZIF‐94‐uIm enhances the CO_2_ permeability, in agreement with the porous structure and CO_2_‐philicity of both MOFs. Notably, the increase in the CO_2_ permeability is comparable for both MOFs, while the selectivity remains similar to that of the pure polymer. These findings demonstrate the amphiphilic nature of ZIF‐94‐uIm, highlighting its excellent compatibility with both hydrophilic (PEBAX 3533) and hydrophobic (PIM‐1) polymeric matrices, even if the combination with PIM‐1 results more advantageous from the point of view of the trade‐off between CO_2_ permeability and CO_2_/N_2_ selectivity (Figure 5a).

## Conclusions

This study investigated the modification of zeolitic imidazolate framework‐94 (ZIF‐94) with 2‐undecylimidazole (umIm) and its integration into mixed matrix membranes (MMMs) based on the polymer of intrinsic microporosity PIM‐1 for gas separation applications. To achieve a superior gas separation performance, both the polymer and nanofiller were precisely engineered to attain the desired properties. The polymer sample selected for this study features a branched topology, optimizing the balance between permeability and selectivity. Meanwhile, the functionalized ZIF‐94‐umIm nanofiller leverages its CO_2_‐philic nature while ensuring a uniform dispersion within the polymer matrix. The characterization techniques and gas separation tests demonstrated that the hydrophobically modified MOF (ZIF‐94‐umIm) exhibits amphiphilic properties and better dispersion and compatibility within the PIM‐1 matrix compared to the original ZIF‐94. MMMs incorporating 5–15 wt.% of ZIF‐94‐umIm were prepared. In contrast, MMMs containing the original ZIF‐94, particularly at loadings exceeding 5 wt.%, exhibited increased rigidity due to poor compatibility, leading to structural damage that made them crack and impeded gas separation measurements. The improved CO_2_ separation performance of MMMs is attributed to an enhanced diffusivity for this molecule, driven by the CO_2_‐philic nature of the material, along with the formation of shorter molecular pathways through the ZIF porosity and the polymer/nanofiller interface. Our findings indicate that incorporating the amphiphilic ZIF‐94‐umIm enhances the CO_2_ permeability by 70% and the CO_2_/N_2_ selectivity by 10%, reaching values of 8900 Barrer and 20.2, respectively. Additionally, the prepared TFNs fall within the required industrial standards, highlighting their potential for advanced CO_2_ capture and gas separation. Overall, this research underscores the significance of tailored nanofillers in optimizing MMM properties and advancing membrane‐based separation technologies for sustainable industrial applications.

## Conflict of Interests

The authors declare no conflict of interest.

## Supporting information



Supporting Information

## Data Availability

The data that support the findings of this study are available from the corresponding author upon reasonable request.
