# Supplementary material for: Amphiphilic Zeolitic Imidazolate Framework for Improved CO2 Separation in PIM‐1 Mixed Matrix Membranes
Source: Angew Chem Int Ed Engl. 2025 Apr 14;64(22):e202420879. doi: 10.1002/anie.202420879 (PMC12105687; doi:10.1002/anie.202420879)
Supplement: Supplementary file 1 — Supporting Information [file ANIE-64-e202420879-s001.docx]

Supporting information

Amphiphilic zeolitic imidazolate framework for improved CO_2_ separation in PIM-1 mixed matrix membranes

Marta Pérez-Miana,^[a,b]^ José Miguel Luque-Alled,^[a,b]*^ Álvaro Mayoral,^[a]^ Íñigo Martínez-Visus,^[a,b]^ Andrew B. Foster,^[c]^ Peter M. Budd,^[c]^ Joaquín Coronas^[a,b]*^

^a^Instituto de Nanociencia y Materiales de Aragón (INMA), CSIC-Universidad de Zaragoza, Zaragoza, 50018, Spain

^b^Chemical and Environmental Engineering Department, Universidad de Zaragoza, Zaragoza, 50018, Spain

^c^Department of Chemistry, School of Natural Sciences, The University of Manchester, Manchester, M13 9PL, UK

*Corresponding author: Joaquín Coronas ([coronas@unizar.es](mailto:coronas@unizar.es)); Jose Miguel Luque-Alled ([jose.luque@unizar.es](mailto:jose.luque@unizar.es))

1. **Experimental section**
   1. **Synthesis of branched PIM-1**

The polymer synthesis procedure employed to obtain a branched PIM-1 polymer, B-PIM-1, is collected in the following sections.

- - 1. **Polymer materials**

The monomers, 5,5′,6,6′-tetrahydroxy-3,3,3′,3′-tetramethyl-1,1′- spirobisindane (TTSBI) and tetrafluoroterephthalonitrile (TFTPN, 100 %) were purchased from Alfa Aesar and Fluorochem, respectively. TFTPN was used as received at certified levels of purity, after drying in vacuum overnight at room temperature. TTSBI required further purification before use. TTSBI was stirred in ethyl acetate at a concentration of 1 g mL^-1^ in a round bottom flask under N_2_ and heated to reflux. Ethyl acetate was added in small aliquots until the TTBSI was fully solubilized. While the solution was still under reflux, an equal amount of hexane was added to the solution while hot and further stirred for 10 min. The solution was cooled to room temperature and then cooled to −5 °C, using a water/dry ice mixture for 4 h. The resulting white precipitate was collected via filtration and washed with hexane to remove any traces of ethyl acetate. After drying under nitrogen for 1 h, the white solid was dried under vacuum at room temperature overnight to avoid oxidation, to afford the desired product as a fine white powder.

Anhydrous potassium carbonate (K_2_CO_3_, 99.5%) was purchased from Fisher Scientific Ltd, with the base ground into a fine powder and dried in a vacuum oven at 110 °C overnight before use. Anhydrous dimethylacetamide, (DMAc), toluene, methanol, chloroform, tetrahydrofuran and 1,4-dioxane were purchased from Sigma-Aldrich and used as received.

- - 1. **Polymer synthesis (B-PIM-1)**

(Significantly branched PIM-1 samples (>10 %) are typically termed as B-PIM-1)

Equimolar amounts of the monomers, TFTPN (100 %, 10.00 g, 0.05 mol) and TTSBI (100 %, 17.02 g, 0.05 mol) were placed along with potassium carbonate (20.73 g, 0.15 mol) into a 500 mL 3-necked, round bottom flask. The solvent mixture of dimethylacetamide (DMAc, 120 ml) and toluene (60 ml) was then added. This equates to an initial 20 vol. % excess of solvent at the start of the reaction compared to the conventionally reported synthesis conditions.^[1,2]^ Both the larger scale of the reaction and the presence of extra solvent mean that the early stages of the polymerization proceed at a lower temperature profile, which encourages more monosubstituted oligomeric structures within the mixture. The flask was equipped with a nitrogen inlet, coil condenser and a Heidolph mechanical stirrer (complete with digital rpm and torque reading display) which was used to mix the reaction mixture. A strong positive pressure of nitrogen was maintained over the reaction mixture. Heating was supplied via a hotplate equipped with a DrySyn aluminium heating block, with the flask inserted together with a temperature probe. The reaction mixture was heated from room temperature to 160 °C, initially stirred at 250 rpm, with the stirring rate increased or decreased as appropriate with changing viscosity of the overall mixture and torque readings recorded at regular intervals. After 15 min, an extra batch of DMAc (20 mL) and toluene (10 mL) solvent mixture was added to the flask to reduce the viscosity of the reaction mixture. This solvent batch addition was repeated after 22 min. After 40 min, the reaction mixture was quenched into excess methanol. The polymer yield obtained after purification (see Section S1.2.3) was 21.9 g (95 %). Molar mass, *M*_w_ = 69,900 g mol^-1^, *M*_n_ = 34,700 g mol^- 1^ (*Đ* = 2.1); ^1^H NMR (500 MHz, CDCl_3_, ppm) δ: 7.58 ppm hydroxyl proton, 6.81, 6.67, 6.42, 6.25 ppm aromatic protons (labelled a, c, b, d in Figure S1), 4.01, 3.93, 3.85 ppm hydroxyl protons, 2.29, 2.15 ppm methylene protons, 1.37, 1.31 ppm methyl protons, full ^1^H NMR spectrum presented in Figure S2; Elemental analysis (EA): C = 73.79 %, N = 6.16 %, H = 4.36 %, F = 0.03 %, predicted EA for disubstituted PIM-1 structure: C = 75.64 %, H = 4.38 %, N = 6.08 %.; The amount of colloidal network content determined by filtration was 10 %.

- - 1. **Purification of polymer**

The recovered filtered polymer was re-dissolved in CHCl_3_ (concentration of 5 g in 120 mL) and re-precipitated by pouring slowly into excess methanol. The polymer was collected via filtration and refluxed in de-ionized water for 16 h. After refluxing in water, the mixture was filtered by vacuum filtration and then immersed in a minimal amount of 1,4-dioxane for 15 min (volume used was just enough to cover the mass of polymer in the beaker) to remove low molar mass oligomers. The polymer was vacuum filtered before washing with excess of acetone to remove traces of 1,4-dioxane. The filtered polymer was then soaked in methanol for 12 h to remove all traces of dioxane and acetone. Finally, the polymer was filtered dry using a sintered funnel under vacuum, before drying in a vacuum oven at 120 °C for 72 h to remove all trace solvents.

- - 1. **Polymer characterization**

Size exclusion chromatography (SEC) analysis: Average molar mass of the polymer was measured by triple detector size exclusion chromatography (SEC). Analysis was performed in chloroform from 1 mg mL^-1^ polymer solution (injection volume 100 µL) at a flow rate of 1 mL min^−1^ using a Viscotek VE2001 SEC solvent/sample module with two PL Mixed B columns maintained at 35 °C and a Viscotek TDA 302 triple detector array (refractive index, light scattering, viscosity detectors). The data was analyzed in OmniSec software.

Nuclear Magnetic Resonance (NMR) analysis: ^1^H NMR spectrum of the polymer was recorded using a Bruker Avance II 500 MHz instrument. 50 mg mL^-1^ polymer solution in CDCl_3_ was prepared for the NMR analysis. Signal peaks for the solvent were used as references. The NMR spectrum obtained for the branched PIM-1 polymer sample is provided in Figure S2. The main aromatic peaks, a and b, associated with di-substituted PIM-1 residue structures are present at 6.81 and 6.42 ppm, respectively. The partially resolved aromatic peaks, c and d, attributed to a branched structure (Figure S1) are present at 6.67 and 6.25 ppm, respectively. The peaks associated with methylene protons, e, in the spiro center are present at 2.15 and 2.33 ppm. The four sets of methyl protons, f, are present at 1.31 and 1.36 ppm. Lorentz peak fitting of the aromatic proton region (δ = 6.0−7.2 ppm) of the proton NMR spectrum was used to determine the respective integral areas associated with resonances attributed to disubstituted PIM-1 residue and branch point stuctures (aromatic protons labelled a, b, c and d in Figure S1). Example of the peak fitting obtained for the aromatic proton region of ^1^H NMR spectrum of B-PIM-1 polymer, is presented in Figures S3. This allowed an estimation of the percentage of branch points present in the PIM polymer sample as a proportion of all residues present.

Determination of network content: A polymer solution in chloroform (1 mg mL^- 1^) was accurately prepared from 10-15 mg of a polymer sample. The entire solution was passed through a 0.45 μm PTFE syringe filter and the exact weight of solution collected into a 30 mL sample bottle was recorded. The solvent in the bottle was allowed to slowly evaporate over several days. Once the solvent had visually completely evaporated, the bottle was placed in an oven at 100 °C to complete the drying process. The weight of polymer remaining in the bottle was measured and compared against the mass of filtered solvent (volume of chloroform) initially collected to determine the filtered polymer concentration. The network content by filtration was determined as the difference between the initial and filtered concentrations of the polymer as a percentage.

Elemental analysis: A Flash 2000 Organic Elemental Analyser (Thermo Scientific, The Netherlands) was employed to obtain elemental analysis (C, H, N) data. 1-2 mg of polymer film was used for each experiment. Fluorine (F) analysis of some polymer samples was carried out by Exeter Analytical UK Ltd. It was carried out by combustion of an accurately weighed sample in a silica oxygen flask over an accurately measured volume of water and total ionic strength adjustment buffer (TISAB) solution. A fluoride ion selective electrode was used to measure the potential of a range of fluoride solutions. The potential of the combusted samples was measured under the same conditions.

Figure S1. Synthesis of PIM-1 from 5,5′,6,6′-tetrahydroxy-3,3,3′,3′-tetramethyl-1,1′- spirobisindane (TTSBI) and tetrafluoroterephthalonitrile (TFTPN), showing ideal disubstituted structures and branched structures that may form as a result of monosubstitution.

Figure S2. ^1^H NMR spectrum of B-PIM-1 polymer (M_w_ = 69,900 g ‧ mol^-1^, Đ = 2.1) synthesized in DMAc/toluene (20 vol % excess at start) with extra solvent (40 vol % more, added after 15 and 22 min respectively) added during the polymerization (0.05 mol scale).

Figure S3. Lorentz peak fitting of aromatic proton region of ^1^H NMR spectrum of B-PIM-1 polymer (M_w_ = 69,900 g ‧ mol^-1^, Đ = 2.1).

- 1. **Synthesis of ZIF-94**

The synthesis of ZIF-94 followed the procedure reported by Paseta et al.^[3]^ Zinc acetate dihydrate (1.584 g, 7.2 mmol) was dissolved in 6 mL of methanol, while 1.584 g (14.4 mmol) of 4-methyl-5-imidazolecarboxaldehyde was dissolved in 15 mL of THF in a separate vial. NaOH was added at a ratio 2:1 (NaOH:Zn molar ratio) to the zinc acetate solution and mixed for 10 min. The resulting mixture was then combined with the ligand dissolution and stirred at room temperature for 1 h. Then, the product was collected and washed once with methanol by centrifugation (Beckman Coulter Allegra x-30) at 10,000 rpm during 10 min at room temperature. Finally, ZIF-94 nanocrystals were activated by refluxing in 100 mL of EtOH for 6 h, followed by centrifugation at 10,000 rpm for 10 min and drying overnight at room temperature.

- 1. **Ligand exchange procedure**

Solvent-assisted ligand exchange (SALE) was performed on ZIF-94 (see scheme in Figure 1a) following our previous work related to the partial substitution of the ligand 2-umIm with the ligand 2-methylimidazole present in ZIF-8.^[4]^ ZIF-94 (0.5 g, 1.75 mmol) previously synthesized was suspended in 42 mL of MeOH. In a separate flask, 2-undecylimidazole (0.779 g, 3.5 mmol, TCI) was dissolved in another 42 mL of MeOH. Both flasks were separately stirred during 10 min at room temperature and then mixed under stirring for 5 min at room temperature. Then, it was poured into a Teflon-lined stainless-steel autoclave, sealed and placed in an oven at 70 °C for 7 and 14 days. Then, the product was centrifuged at 9000 rpm for 15 min, washed three times with MeOH and dried overnight at 60 °C under vacuum. The obtained samples, denoted as ZIF-94-umIm (7d) and ZIF-94-umIm (14d), exhibit an amphiphilic nature. Both are composed of a hydrophilic component (the aldehyde group from the non-substituted mImca) that will be CO_2_-philic and a hydrophobic part (2-undecylimidazole) that would be more compatible with PIM-1 polymer (see scheme in Figure 1b).

- 1. **Preparation of PIM-1 membranes and mixed matrix membranes (MMMs)**

PIM-1 powder (0.075 g) was dissolved in chloroform (1.87 mL) under stirring during 2 h at room temperature. PIM solution was filtered with a PTFE 0.45 µm filter and MOF powder (5, 10 and 15 wt. % loading) was dispersed into it, alternating 3 cycles of 30 min of stirring and sonicating. After achieving an adequate dispersion of the MOFs, the casting suspensions were carefully poured onto PTFE Petri dishes and allowed to evaporate over 48 h, resulting in membranes with a thickness of ca. 50 µm. Afterwards, the membranes were peeled off, immersed in MeOH for 1 h, then they were soaked again in fresh MeOH for 6 h more and dried under vacuum in an oven at 60 °C overnight. For the fabrication of MMMs, only ZIF-94 and ZIF-94- umIm (14d), were used. The MOF content in the MMMs was expressed as wt.%, for example, 5% ZIF-94-umIm corresponds to a 5 wt.% loading of ZIF-94-umIm (14d) in PIM-1.

- 1. **Preparation of PEBAX membranes and mixed matrix membranes (MMMs)**

Polyether-block-amide, specifically Pebax® 3533 SA 01 MED, which consists of 75 wt. % poly(tetramethylene oxide) (PTMO) and 25 wt. % aliphatic polyamide (PA12), was supplied in pellet format by Arkema, France. Pebax® 3533 pellets were initially dissolved in a 3:1 (v/v) mixture of 1-propanol and 1-butanol. The mixture was stirred under reflux at 80 °C for 2 hours to prepare a 3 wt. % casting solution. After cooling down, the solution was poured onto a glass Petri dish and allowed to evaporate overnight in an oven at 40 °C. To fabricate MMMs, once the pellets were dissolved under reflux, ZIF-94 and ZIF-94-umIm (14d) were added as filler at different loadings (5 and 10 wt.%). Suspensions were sonicated and stirred three times for 30 minutes each step. Finally, the dispersions were poured onto a glass Petri following the same conditions as pristine Pebax membranes.

- 1. **Preparation of Thin-film nanocomposite (TFN) membranes**

TFN membranes were composed of a Poly(vinylidene fluoride (PVDF) porous support, an intermediate Poly(1-trimethylsilyl-1-propyne) (PTMSP) layer, and a selective PIM-1 film incorporating ZIF-94-umIm nanoparticles. The porous support was fabricated by phase inversion process, as detailed in Alberto et al.,^[5]^ with a modified casting solution consisting of 18 wt. % PVDF (M_w_ = 530 000 g ‧ mol^-1^), 1 wt. % H_3_PO_4_, and 2 wt. % LiCl. The PTMSP layer, acting as a gutter layer, was applied suing spin coating at 4000 rpm and 20 seconds, and employing a solution of 2 wt.% of PTMSP in hexane. The selective layer was similarly produced by spin coating at 8000 rpm and 20 s using a 3 % (w ‧ v^-1^) PIM-1 solution in CHCl_3_, with ZIF-94-umIm (14d) dispersed at a concentration of 5 wt. % relative to the polymer.

- 1. **Characterization**

Thermogravimetry analyses (TGA) were conducted using Mettler Toledo TGA/STDA 851e. Samples were placed in 70 µL alumina pans and heated under airflow from 35 to 700 °C at a heating rate of 10 °C ‧ min^-1^.

X-ray diffraction (XRD) measurements were performed using a Panalytical Empyrean instrument with CuKα radiation (λ = 0.154 nm), scanning over the 2θ angle range of 5 - 40 ° at a rate of 0.03 ° ‧ s^-1^.

Fourier transform infrared spectroscopy with attenuated total reflection (FTIR-ATR) was carried out using a Bruker Vertex 70 FTIR spectrometer equipped with a DTGS detector and a Golden Gate diamond ATR accessory. Spectra were recorded by averaging 16 scans in the wavenumber range of 4000-600 cm^−1^ at a resolution of 4 cm^−1^.

Scanning electron microscopy (SEM) imaging of the MOFs and membranes was performed using an Inspect F50 model scanning electron microscope (FEI) operated at 10 kV and coating samples with Pd. Membrane cross-sections were prepared by cutting after immersion in liquid N_2_. The microscope is equipped with an energy-dispersive X-ray detector used for EDS analysis.

CO_2_ adsorption-desorption isotherms were obtained using a Micromeritics ASAP 2020 at 273 K. Prior to measurements, two degasifications were performed at 200 °C with a heating rate of 10 °C ‧ min^-1^ under vacuum for 8 h. Brunauer-Emmett-Teller (BET) analysis was employed to calculate the specific surface area of porous materials. The Horvath-Kawazoe (HK) model was employed to analyse the MOF pore size, assuming cylindrical pores.

Water contact angle (WCA) measurements were conducted using a Krüss Drop Shape Analyzer 10 MK2 at room temperature, with measurements taken in at least 6 areas of each membrane, using a volume drop of 4 µL.

Elemental analysis was performed in a Perkin Elmer Series II 2400 CHNS/O Analyzer. It was measured using the CHNS method without optimizing the oxygen input; that is, without providing extra seconds of oxygen flow for enhanced combustion. Weighing was conducted at room temperature with exposure to air until it was encapsulated in tin capsules. The microbalance used is the Provectus 6500, also from Perkin Elmer, and it is connected to the analyzer.

Viscosity tests were carried out with a SMART L Fungilab Rotational viscometer. Casting solutions (6.5 mL) placed in an APM/B adapter were exposed to different rotational speeds (from 30 to 200 rpm) at 22 °C with a TL5 spindle. At rotational speeds above 150 rpm, the measurements were obtained within the recommended torque of the viscometer company. The measurements were performed in casting solutions of pristine PIM-1 (4 w ‧ v^-1^ % of polymer in CHCl_3_), PIM-1 with ZIF-94 (at the same polymer concentration and 10 wt.% of ZIF loading) and with ZIF-94-umIm (the same polymer and ZIF concentration).

Dynamic light scattering (DLS) measurements were obtained using BI-90 Plus Brookhaven Particle Size Analyzer. The hydrodynamic diameter of both original ZIF-94 and modified version was measured in suspensions of 1.67 mg ZIF/mL CHCl_3_. Measurements of both suspensions were conducted within 20 min of their preparation.

Mechanical properties of membranes were determined by performing uniaxial tensile tests utilizing the normal force measurement capabilities of a MCR 702e rheometer (Anton Paar). Sample specimens were rectangular strips of approximately 20-25 mm length, width of 5 mm and thickness of about 40 µm. Specimens were clamped using the solid rectangular fixture of the instrument. The initial free length between clamps varied according to the individual specimen length when mounted and was measured by the instrument. Likewise, for each specimen the individual width and thickness was measured. Elongation rate of 1 mm min^−1^ was set. Tensile tests were performed at ambient temperature. Engineering strain was used for the determination of the E-modulus. The reported values were the average of at least two different membrane samples.

High resolution-transmission electron microscopy (HR-TEM) images of ZIF nanoparticles were obtained using a FEI Titan XFEG operated at 300 kV, fitted with a spherical aberration corrector for the electron probe. TEM specimens were prepared by depositing three small droplets of the ZIF suspensions in ethanol onto a holey carbon copper grid and left to dry. HR-TEM images of thin-film nanocomposite membranes were acquired using a Thermo Fisher Scientific Tecnai F30 microscopy, fitted with a High-Angle Annular Dark Field (HAADF) detector for scanning transmission electron microscopy (STEM) mode and an energy dispersive X-ray spectroscopy (EDS) detector. Ultrathin TEM specimens, approximately 70 nm thick, were prepared by ultramicrotomy (Leica EM UC7 ultramicrotome). The TFN membranes were immersed in EMBed 812 epoxy resin under vacuum overnight at room temperature, followed by curing at 60 ºC for 48 h. The resin-embedded samples were initially trimmed with a glass knife and then sectioned using a diamond knife set at a 6º angle and operating at a speed of 1 mm ‧ s^-1^. The ultrathin sections were retrieved from DI water and transferred onto 300-mesh copper grids with a carbon support layer

- 1. **Gas separation performance**
     1. **Mixed gas testing using He as a sweep gas in the downstream side**

For gas separation measurements, membranes were placed in a gas separation membrane module comprising two stainless steel pieces and a macroporous disk support made of 316L stainless steel (Mott Co.) with a 20 µm nominal pore size and a circular area of 2.12 cm^2^. Membranes were held inside the module using Viton o-rings. The gas separation measurements were conducted by introducing CO_2_/N_2_ (15/85, v/v) mixtures and CO_2_/CH_4_ (50/50, v/v) mixtures to the upstream (or feed) side at an operating pressure of 3 bar (pressure differences of 2 bar, where the upstream side is maintained at 3 bar and the downstream side at 1 bar). Mass-flow controllers (Alicat Scientific, MC-100CCM-D) were utilized to ensure steady and controlled gas flows of the studied molecules, 15 and 85 cm^3^(STP) min^−1^ of CO_2_ and N_2_ in the upstream side, respectively. The downstream (or permeate) side of the membrane was swept at atmospheric pressure (~ 1 bar) with a 50 cm^3^(STP) min^−1^ of He. Concentrations of CO_2_, N_2_ and CH_4_ in the downstream side were analyzed by an Agilent 3000 A micro-gas chromatograph. Permeability was quantified in Barrer (1 Barrer = 10^−10^ cm^3^(STP) cm cm^−2^ s^−1^ cmHg^−1^) once a steady-state condition was achieved. The CO_2_/N_2_ and CO_2_/CH_4_ separation selectivities were determined as the ratio of their respective permeabilities. To maintain the experimental temperature at 35 °C, the membrane module was placed in a UNE 200 Memmert oven. The reported permeability and selectivity values represent the average of at least three independent measurements with different membrane coupons.

- - 1. **Mixed gas testing without sweep gas in the downstream side**

To avoid the use of He during testing and enhance comparability with industrial processes, the fabricated TFNs were also evaluated without a sweep (or carrier) gas. The membrane coupons were mounted in the permeation cell as described above. CO_2_/N_2_ (50/50 and 15/85, v/v) mixtures were introduced on the upstream side, applying transmembrane pressures ranging from 3 to 4.5 bar, while maintaining the downstream side at 1 bar. Cross-flow was maintained on the upstream side at 1 ml ‧ min^-1^, with the stage cut kept below 2% to prevent concentration polarization. The overall permeation rate was determined using a constant pressure system. The time required to collect a specific volume of downstream gas was measured and gas permeance was calculated using the following equation:

$$Gas permeance= \frac{Q}{t \cdot A \cdot(P_{1}- P_{2})}$$

where gas permeance is expressed in GPUs (1 GPU = 10^−6^ cm^3^ [STP] ‧ cm^−2^ ‧ s^−1^ ‧ cmHg^−1^ = 3.348 ‧ 10^−10^ mol ‧ m^−2^ ‧ s^−1^ ‧ Pa^−1^), *Q* represents the volume of permeated gas (cm^3^, corrected to STP [0 °C, 1 atm]), *t* is the permeation time (s), *A* is the active membrane area (cm^2^), and *p_1_* and *p_2_* (cmHg) correspond to the pressures on the upstream and downstream sides of the membrane, respectively.

The composition of the permeation gas was quantified using an Agilent 3000 A micro-gas chromatograph without sweep gas, which was used to determine the gas pressure on the downstream side (*p_2_*). The equipment was calibrated for various CO_2_/N_2_ ratios to cover the entire range of analysis, from 100/0 to 15/85.

1. **Results**


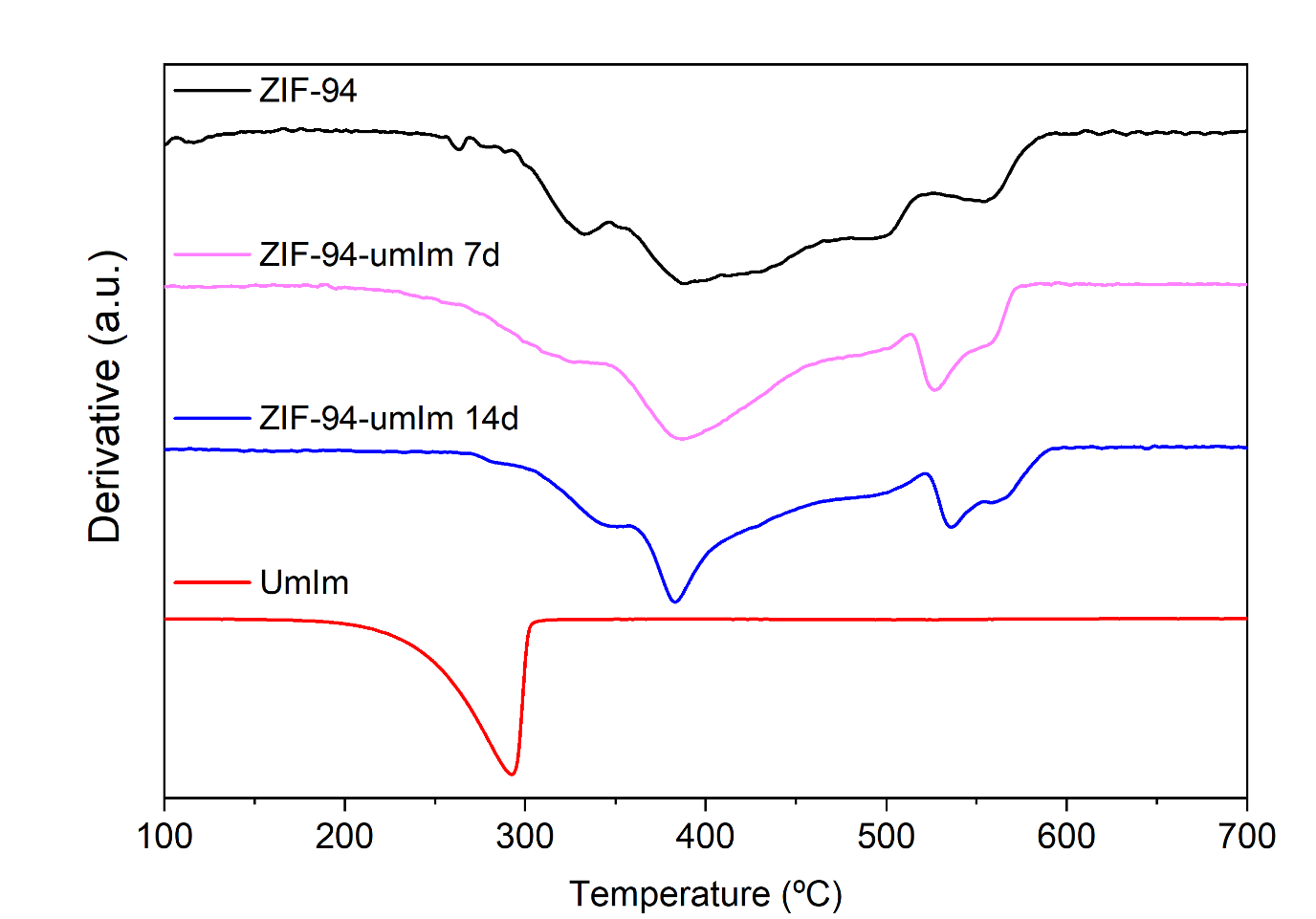


Figure S4. Weight loss derivative vs. temperature of ZIF-94 (black), ZIF- 94 - umIm (7d) (pink), ZIF- 94- umIm (14d) (blue), and pure umIm (red).


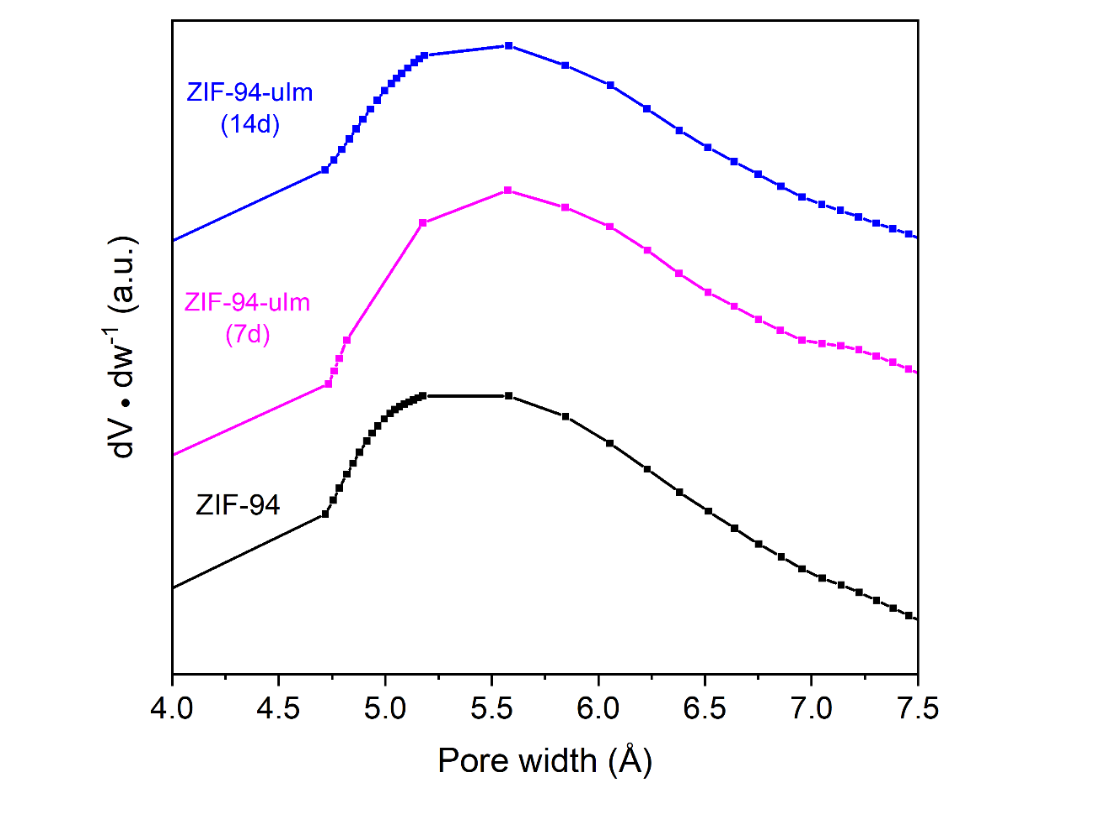


Figure S5. Pore size distributions of ZIF-94 (black), ZIF- 94-umIm (7d) (pink), and ZIF- 94-umIm (14d) (blue) calculated from the CO_2_ adsorption isotherms using the Horvath-Kawazoe model.


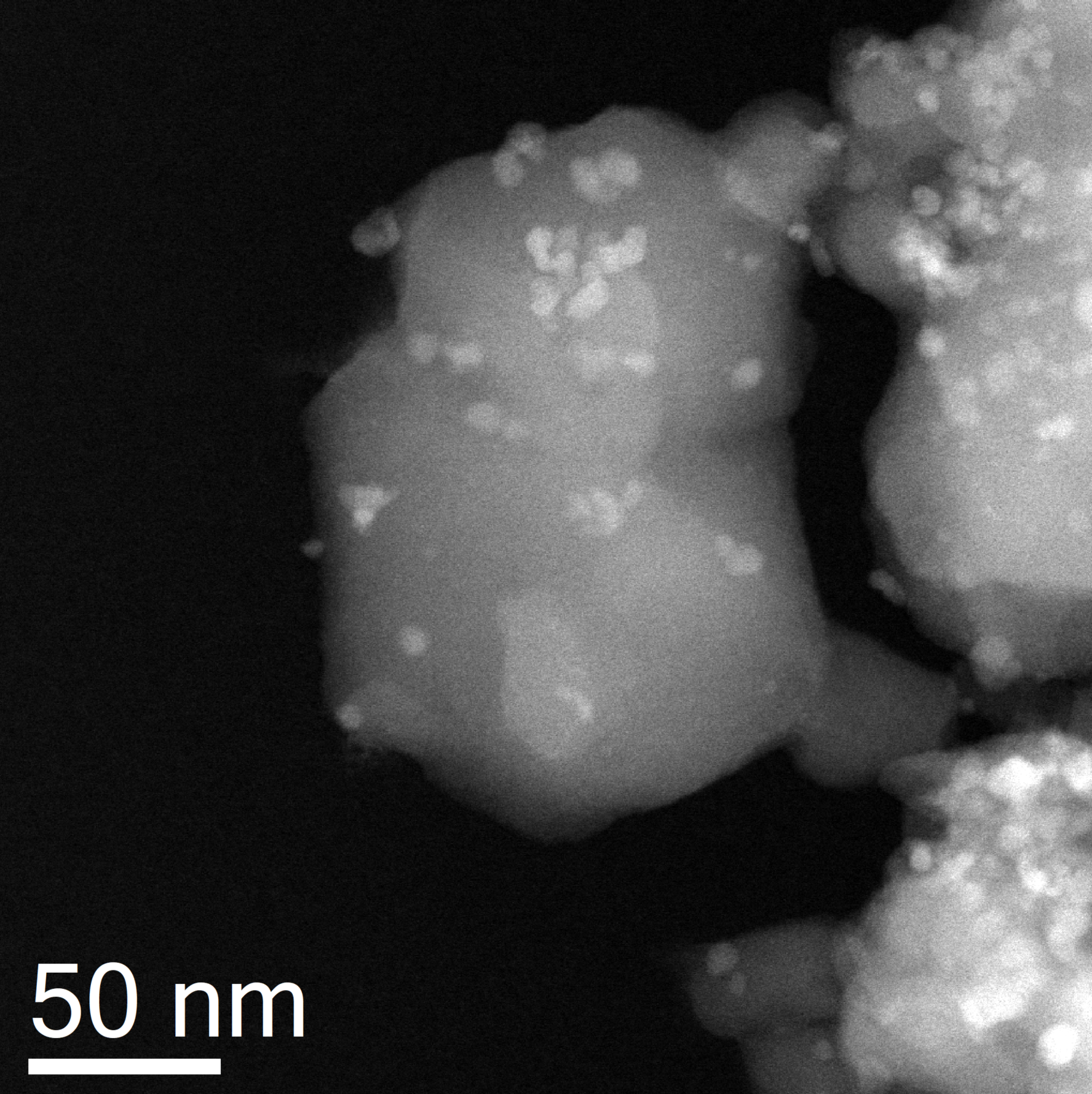


Figure S6. HR-TEM image of ZIF- 94 - umIm (14d).


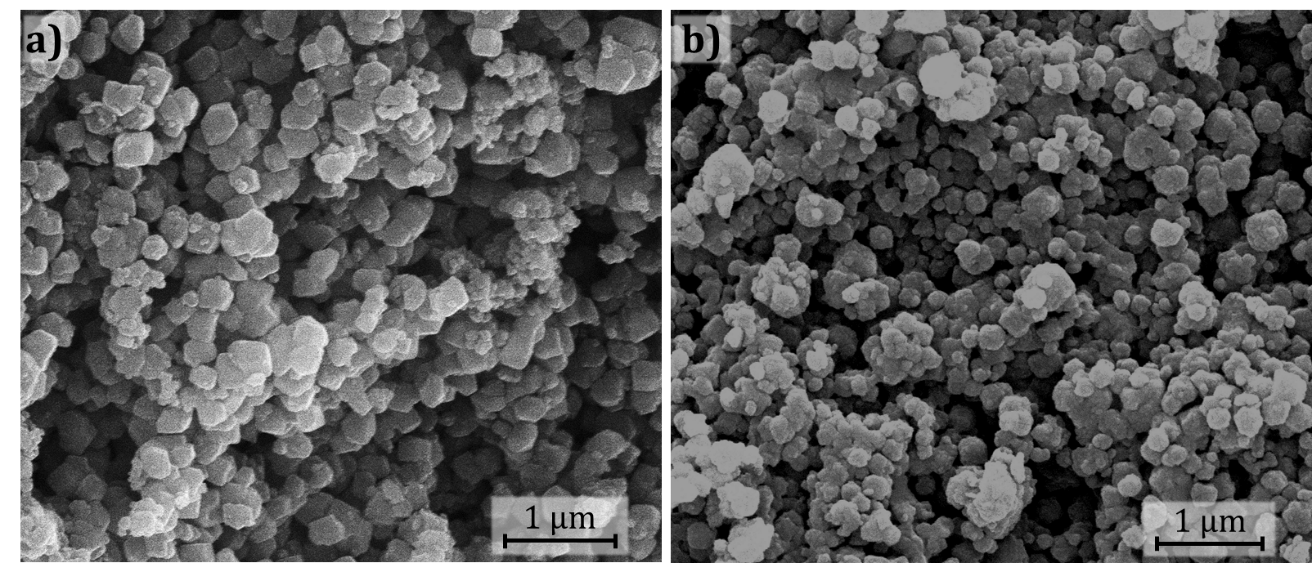


Figure S7. SEM images at the same magnifications (x 50 000) of (a) ZIF-94 and (b) ZIF- 94 - umIm (14d).


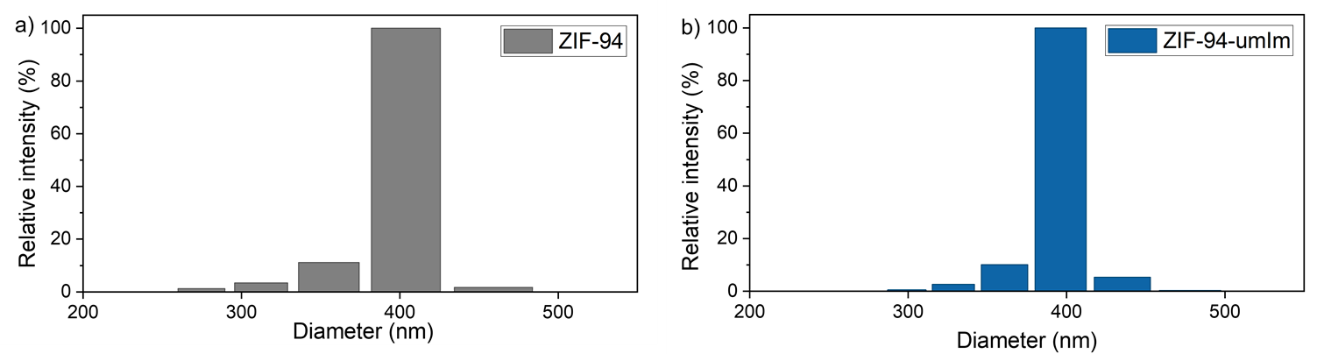


Figure S8. DLS measurements of ZIF-94 (a, grey) and ZIF- 94 - umIm (14d) (b, blue).

Table S1. Content of C, H and N in different samples obtained from elemental analysis and from theoretical calculations.

|  | **C**  (%) | **H**  (%) | **N**  (%) | **Sum**  **(C, H, N)**  (%) | $\frac{\boldsymbol{C}}{\boldsymbol{(C,H,N)}}$  (%) |
| --- | --- | --- | --- | --- | --- |
| **ZIF-94-umIm (14d)** | 42.7 | 4.9 | 15.2 | 62.9 | **67.9*** |
| **ZIF-94**  **(theoretical in Zn(mImca)_2_: 0 % exchange)** | 42.2 | 4.4 | 24.6 | 71.2 | **64.5** |
| **ZIF-94-umIm**  **(theoretical in Zn(umIm)_2_ :100 % exchange)** | 66.2 | 9.9 | 11.0 | 87.1 | **75.9** |

The exchange percentage of substitution was calculated as the variation of the amount of C in base of C, H and N. Once these values were obtained, the exchange was obtained by extrapolating the value obtained from elemental analysis for sample ZIF-94-umIm (14d) (67.9 %), considering the theoretical value of ZIF-94 as 0 % of exchange (64.5 %) and a supposed Zn(umIm)_2_ as 100 % of exchange (75.9 %), obtaining 29.96 % of substitution.

The following equations were used for the calculations:

***Equation S1:***

***% C in CHN =** $\frac{\boldsymbol{\%C}}{\boldsymbol{(\%C+\%H+\%N)}}\boldsymbol{=}\frac{\boldsymbol{42.7}}{\boldsymbol{(42.7+4.9+15.2)}}\boldsymbol{=}$ **67.9 %** C in ZIF-94-umIm (14d)

***Equation S2:***

$$\frac{(67.9-64.5)}{(75.9-64.5)}=\frac{(x-0)}{(100-0)}\to x=\boldsymbol{29.96 \%} of substitution$$

The variations in hydrophobicity/hydrophilicity behavior were investigated. Both ZIF-94 and ZIF-94-umIm (14d) were immersed in a mixture of immiscible solvents (hexane and water). The mixture was shaken until an emulsion formed due to the immiscibility of water and hexane. ZIF-94 particles dispersed in water, while an emulsion formed in the organic phase. In contrast, the modified MOF (ZIF-94-umIm (14d)) formed an emulsion exclusively at the interface between the two solvents, consistent with its amphiphilic property.


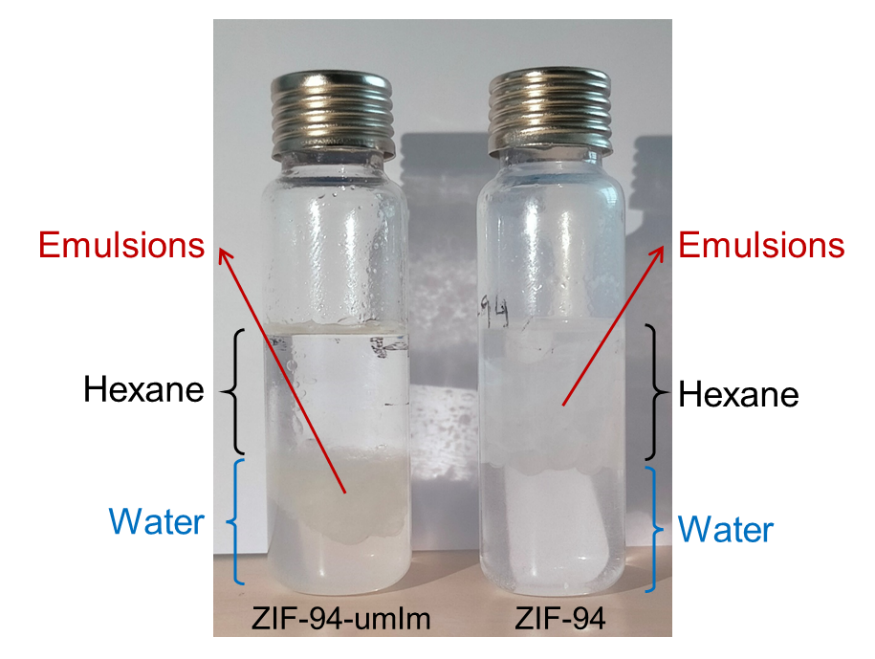


Figure S9. ZIF-94-umIm (left) and ZIF-94 (right) suspensions in hexane:water with their corresponding emulsions in water and hexane, respectively.

Figure S10. Derivative curves of TGA performed in membranes of pristine PIM-1 (green), PIM-1 with ZIF-94 (black) and with ZIF-94-umIm (14d) (blue), both with 10 wt.% loading of filler, and umIm (red).


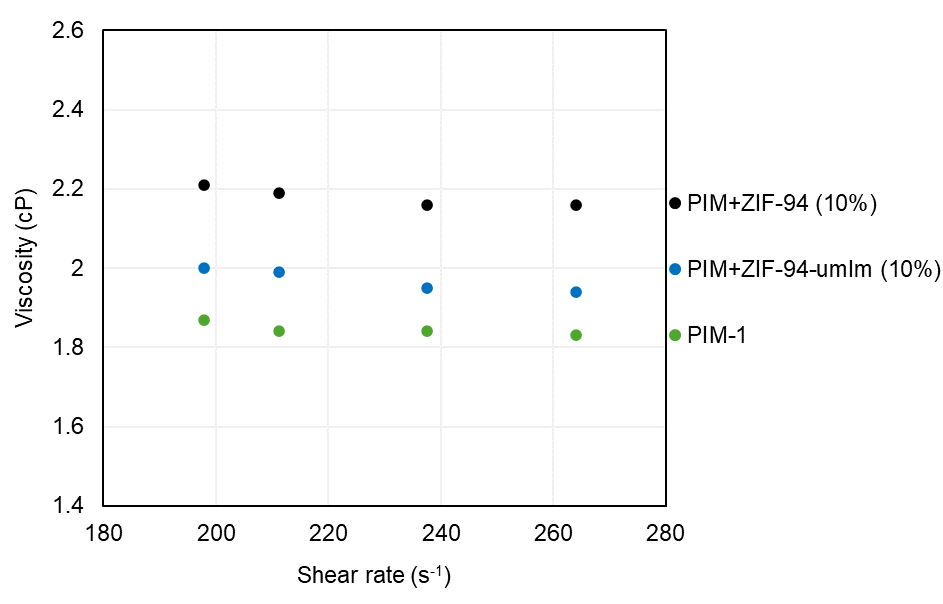


Figure S11. Viscosity of casting solutions of pristine PIM-1 (green), PIM-1 with ZIF-94 (black) and with ZIF-94-umIm (blue), both with 10 wt.% loading of filler.

Table S2. Gas permeability data of different membranes included in Figure 5b.

| **Loading** | **CO_2_ Permeability (Barrer)** | **CO_2_/N_2_ Selectivity** | **Icon** | **Ref.** |
| --- | --- | --- | --- | --- |
| PIM-1 | 5354 | 18.4 | ● | This work |
| 5% ZIF-94-umIm | 8861 | 20.2 | ■ | This work |
| 5% ZIF-94-umIm_Aged (8 m) | 5150 | 23.8 | ■ | This work |
| 10% ZIF-94-umIm | 9216 | 16.5 | ■ | This work |
| 15% ZIF-94-umIm | 8248 | 17.5 | ■ | This work |
| 5% ZIF-94 | 7731 | 18.8 | □ | This work |
| PIM-1 with ZIF-8 | 6300 | 18.0 | *1 | ^[6]^ |
| PIM-1 with ZIF-8-umIm | 16667 | 15.4 | *2 | ^[4]^ |
| PIM-1, 6FDA-DAM and ZIF-8 | 9300 | 14.5 | *3 | ^[7]^ |
| PIM-1 | 13600 | 16.6 | *4 | ^[8]^ |
| PIM-PI-EA | 7340 | 19.9 | *5 | ^[9]^ |
| CPIM- 1 | 18900 | 19.3 | *6 | ^[10]^ |
| CANAL-Me-Me2F | 5400 | 14.6 | ●7 | ^[11]^ |
| PIM-BTrip | 9200 | 27.1 | ●8 | ^[11]^ |
| TTA-100 | 6037 | 24.1 | ●9 | ^[12]^ |
| PIM-1 with 8 % UiO-66-(CF_3_)_2_ | 5242 | 33.8 | *10 | ^[13]^ |
| PIM-1 with polyUiO-66(1:4) | 9659 | 21.5 | *11 | ^[14]^ |
| PIM-1 with 6 % TpTta-COF | 9672 | 26.3 | *12 | ^[15]^ |

Table S3. Gas separation values and score improvement of several MMM containing PIM with different ZIFs as filler.

| **Loading** | **CO_2_ Permeability (Barrer)** | **CO_2_/N_2_ Selectivity** | **Score improvement** | **Ref.** |
| --- | --- | --- | --- | --- |
| 5% ZIF-94-umIm | 8861 | 20.20 | 0.25 | This work |
| 10% ZIF-94-umIm | 9216 | 16.50 | 0.07 | This work |
| 15% ZIF-94-umIm | 8248 | 17.50 | 0.09 | This work |
| 5% ZIF-94 | 7731 | 18.80 | 0.14 | This work |
| 5% ZIF-8 | 19000 | 10.00 | -0.14 | ^[4]^ |
| 5% ZIF-8-umIm | 15582 | 14.50 | 0.15 | ^[4]^ |
| 5% Nanosized ZIF-67 | 3658 | 13.55 | 0.10 | ^[16]^ |
| 10% Nanosized ZIF-67 | 3152 | 15.26 | 0.10 | ^[16]^ |
| 15% Nanosized ZIF-67 | 2567 | 19.89 | 0.16 | ^[16]^ |
| 0.35 % ZIF-8 | 1365 | 25.10 | -0.23 | ^[17]^ |
| 6.5% ZIF-8 | 70 | 21.30 | -1.35 | ^[17]^ |
| 28 % ZIF-8 | 17050 | 15.64 | -0.35 | ^[6]^ |
| 43 % ZIF-8 | 19350 | 11 | -0.64 | ^[6]^ |
| 30 % ZIF-67 | 5206 | 24.2 | 0.22 | ^[18]^ |
| 10 % ZIF-8 | 2891 | 18.1 | 0.01 | ^[7]^ |
| 3 % ZIF-8 | 2586 | 20.1 | 0.03 | ^[7]^ |
| 10 % ZIF-67 | 13255.8 | 9.3 | -0.32 | ^[19]^ |
| 0.5 % TMGHPhO/9.5 % ZIF-67 | 10204.4 | 8 | -0.54 | ^[19]^ |
| 10 % TMGHPhO@ZIF-67 | 7145 | 14 | -0.13 | ^[19]^ |
| 0.5 % TMGHIM/9.5 % ZIF-67 | 9090.5 | 11.9 | -0.21 | ^[19]^ |
| 10 wt % TMGHIM@ZIF-67 | 12848.5 | 13.7 | 0.04 | ^[19]^ |
| 10 % ZIF-71 | 4271 | 19.4 | 0.05 | ^[20]^ |
| 20 % ZIF-71 | 5942 | 20 | 0.19 | ^[20]^ |
| 30 % ZIF-71 | 8377.1 | 18.3 | 0.22 | ^[20]^ |


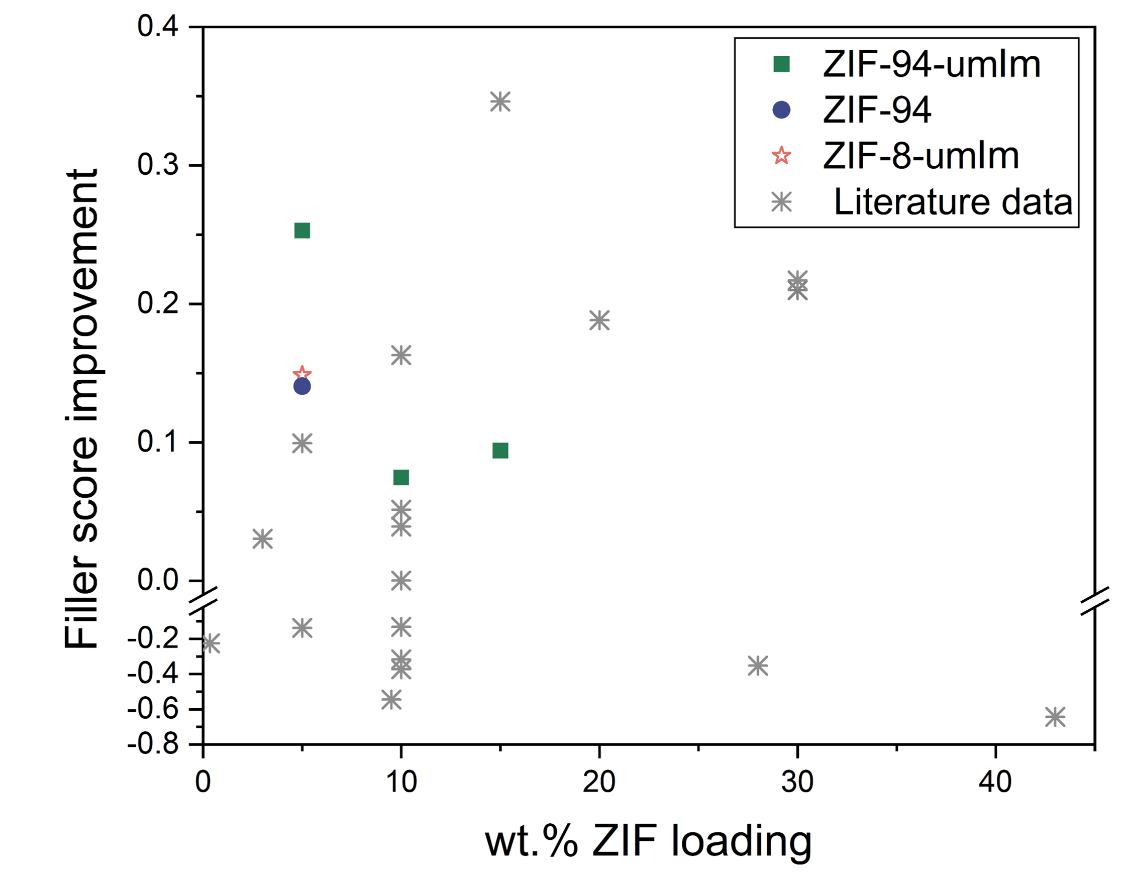


Figure S12. Score improvement for several MMMs composed of PIM-1 and various ZIF nanoparticles, including ZIF-8, ZIF-67, ZIF-71, ZIF-94, and functionalized derivatives of these ZIFs (data are collected in Table S3).


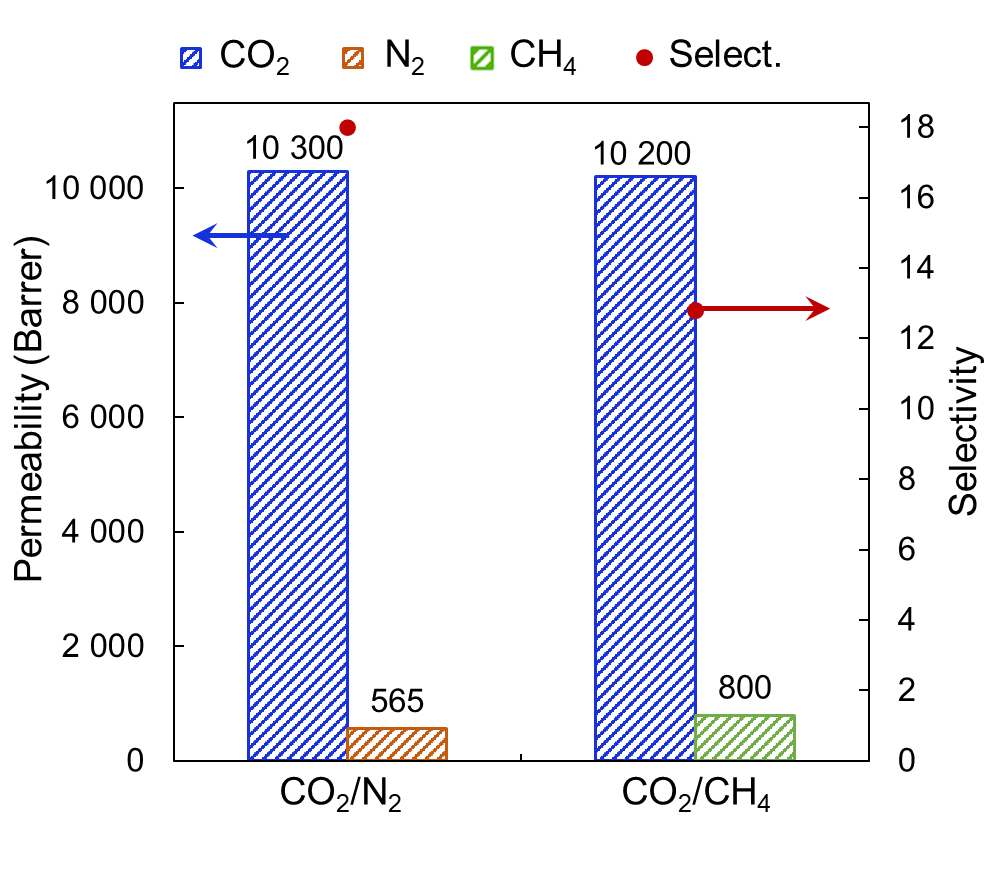


Figure S13. Gas separation performance of membrane with 5 wt.% loading of ZIF-94-umIm for the separation of 50/50 CO_2_/N_2_ and CO_2_/CH_4_.

All membranes exhibit a reduction in the CO_2_ permeability accompanied by a decrease in the CO_2_/N_2_ selectivity for both PIM-1 and MMMs containing ZIF-94-umIm. As CO_2_ sorption sites are deployed, competitive sorption behavior is affected, resulting in a lower separation selectivity. However, MMMs containing ZIF-94 display a slight increase in selectivity at 3 and 4 bar, which may be attributed to the additional sorption sites provided by the CO_2_-philic MOF


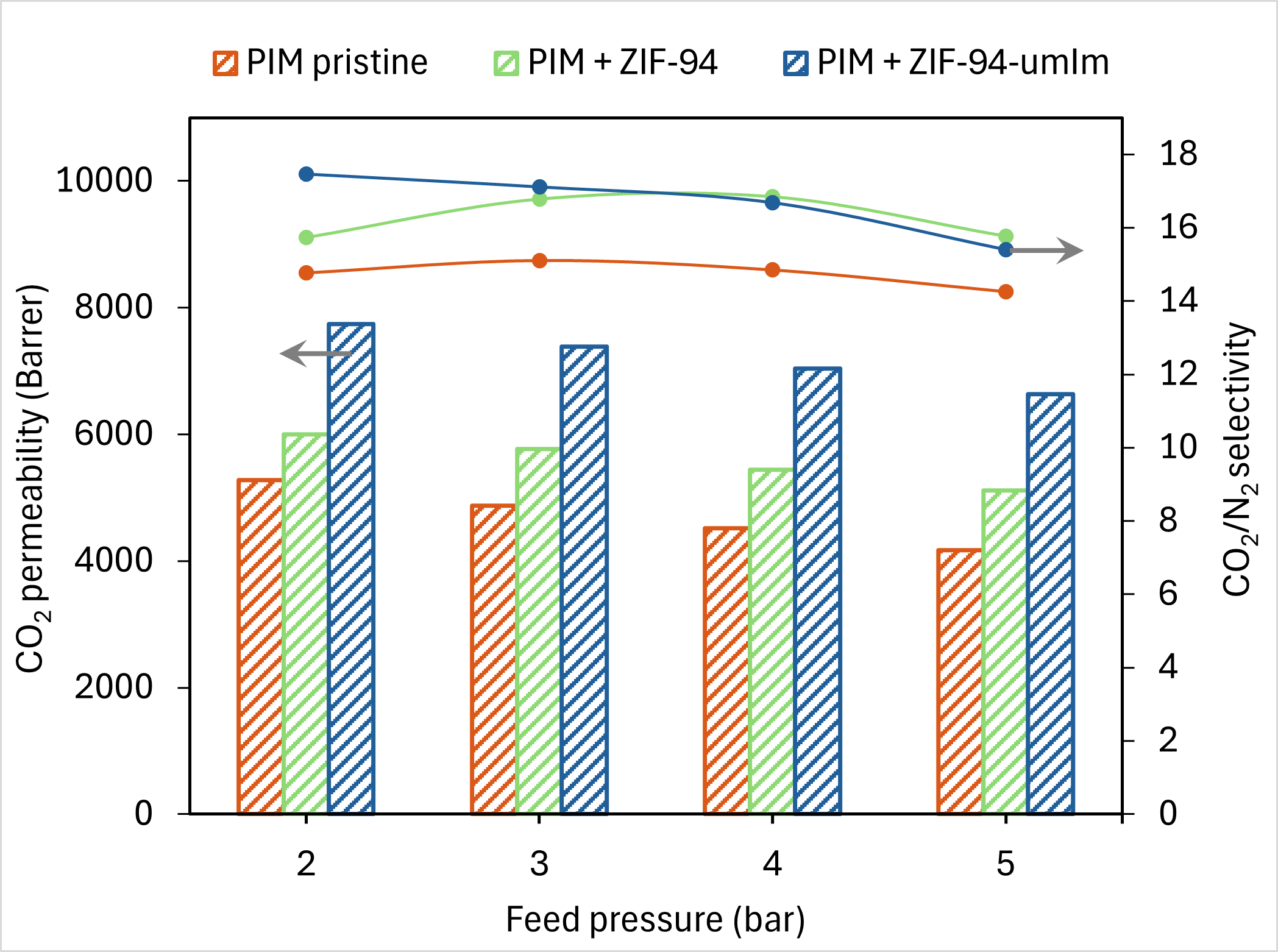


Figure S14. CO_2_ permeabilities and CO_2_/N_2_ selectivities at different upstream pressure (1-5 bar) for pure PIM-1 and MMMs containing 5 wt.% of ZIF-94 and ZIF-94-umIm.


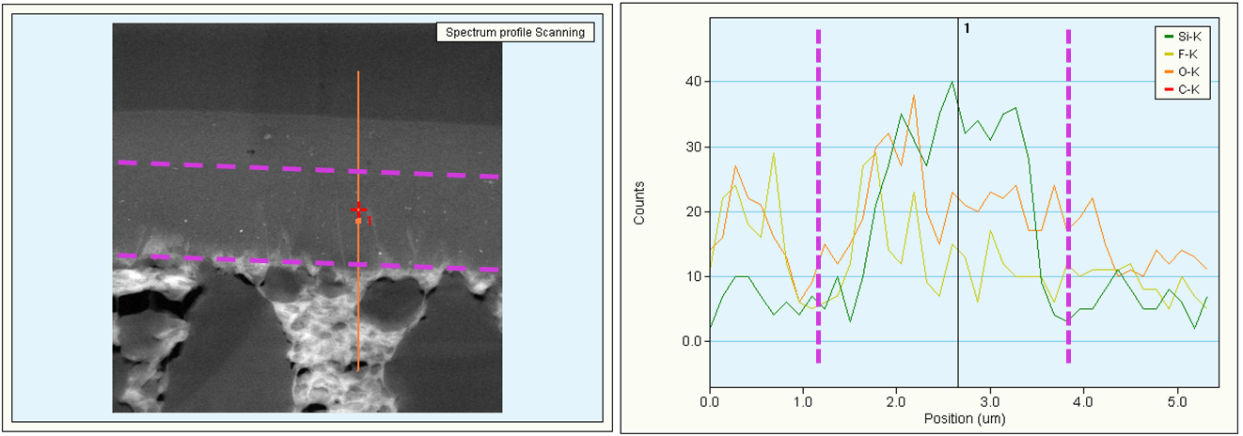


Figure S15. STEM-HAADF image of a cross-section of a TFN membrane accompanied by the corresponding EDX line spectrum. Dashed violet lines indicate the interphase between layers.


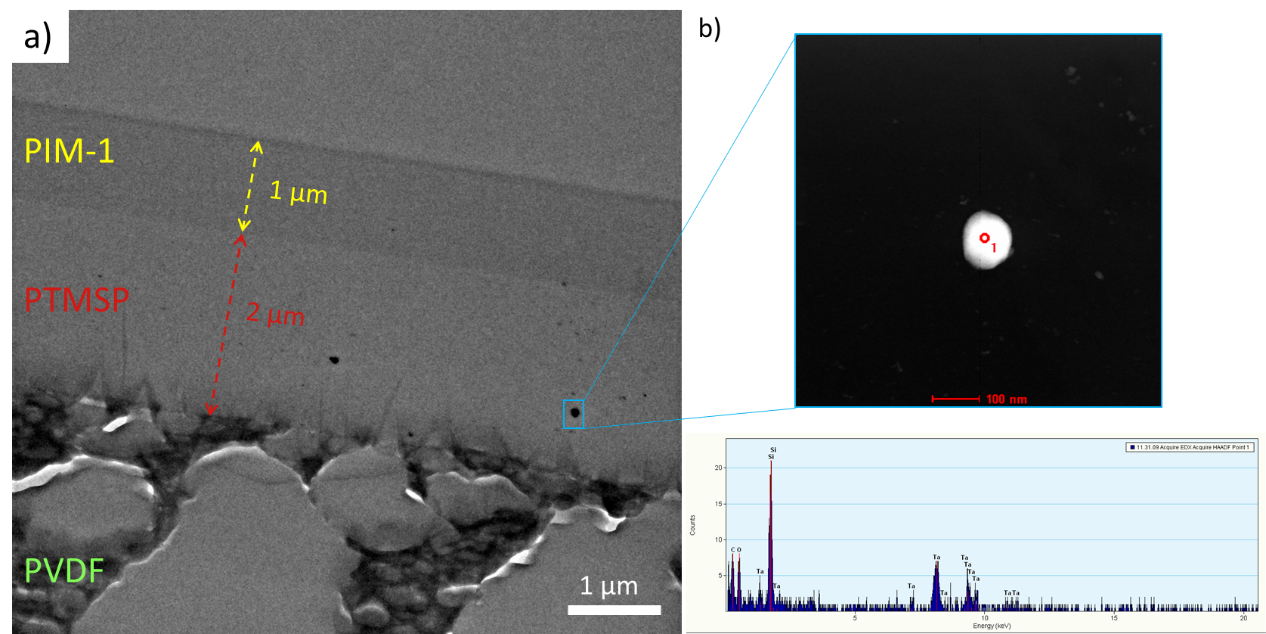


Figure S16. (a) HR-TEM image of a cross-section of a TFN membrane and a (b) STEM-HAADF image with EDX analysis showing well-defined spheres identified as tantalum impurities from the sample preparation process.


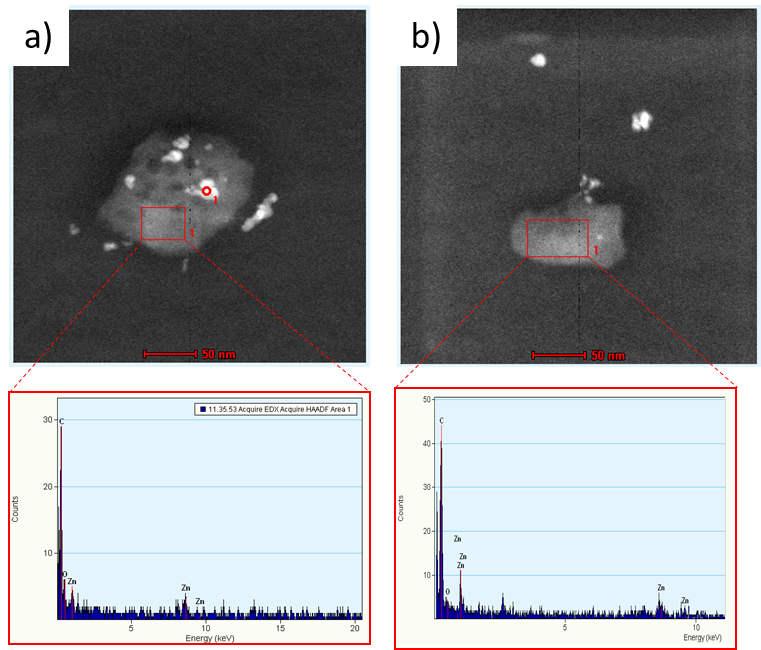


Figure S17. (a and b) STEM-HAADF images of ZIF-94-umIm nanoparticles accompanied by their corresponding EDX line spectrum.


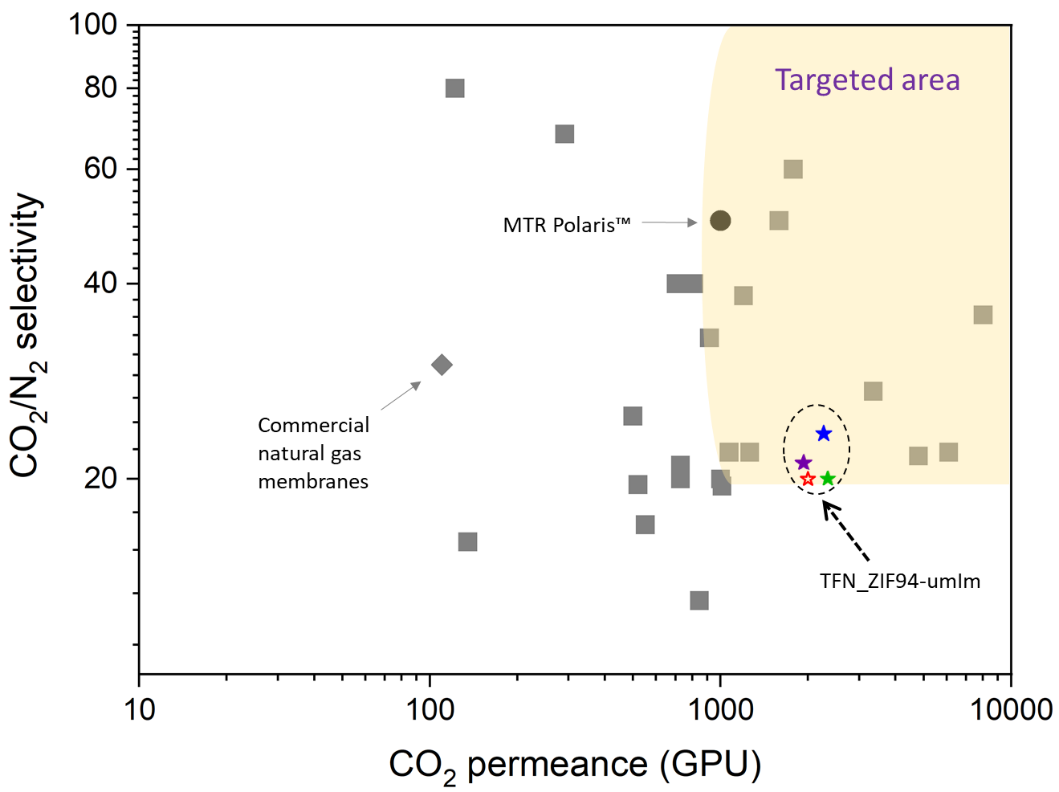


Figure S18. Double-logarithmic plot of CO_2_/N_2_ selectivity versus CO_2_ permeability, comparing literature data^[14,15,21,22]^ with TFN membranes developed in this study (colored stars):
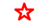
: 2 bar, 50:50 (CO_2_/N_2_) feed side, measured with helium as a carrier gas;
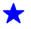
: 2 bar, 50:50 (CO_2_/N_2_) feed side, measured without carrier gas;
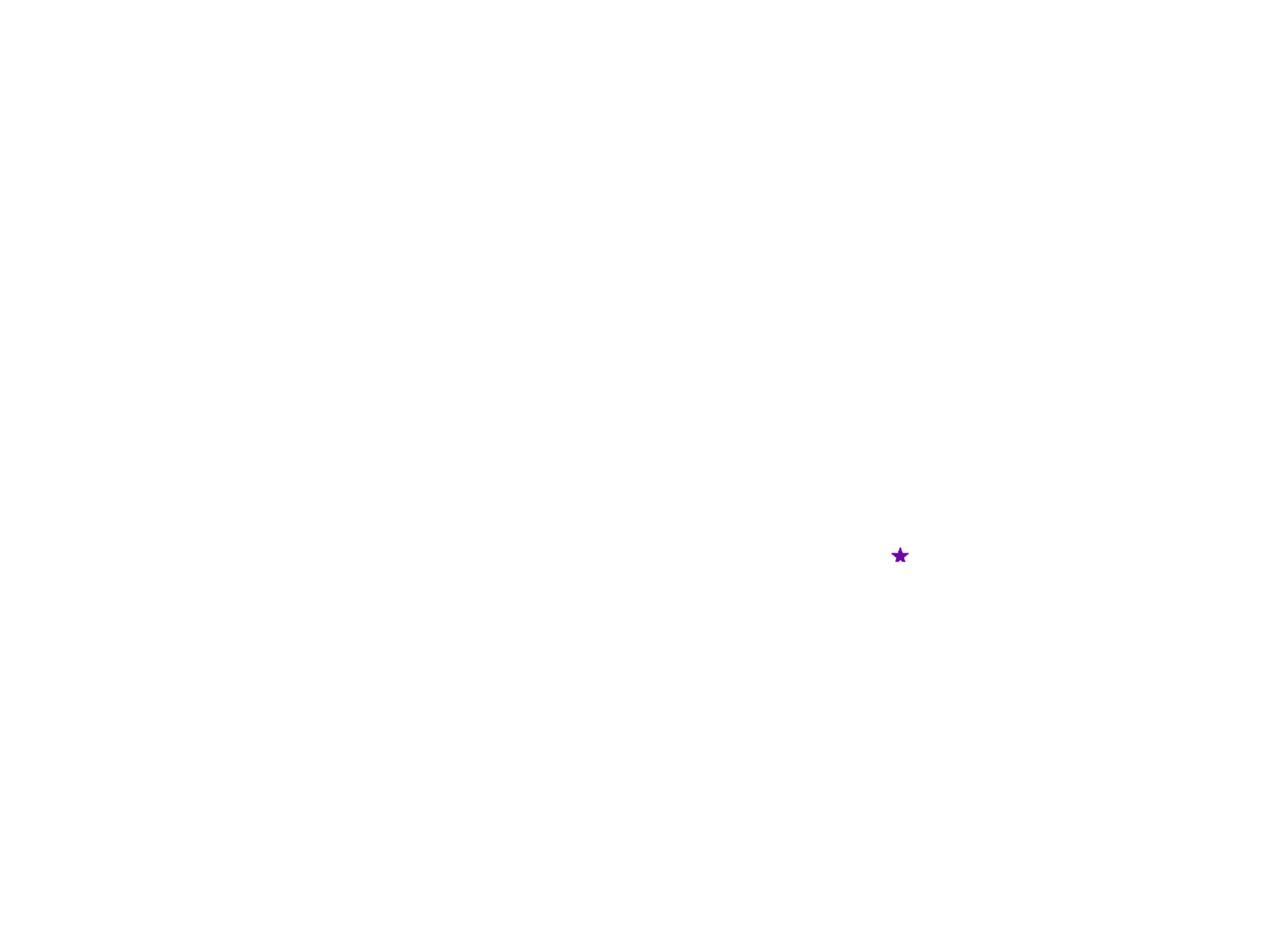
: 3.5 bar, 50:50 (CO_2_/N_2_) feed side, measured without carrier gas;
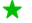
: 2 bar, 15:85 (CO_2_/N_2_) feed side, measured without carrier gas. The yellow area represents the designated target zone for industrial applications.^[21]^


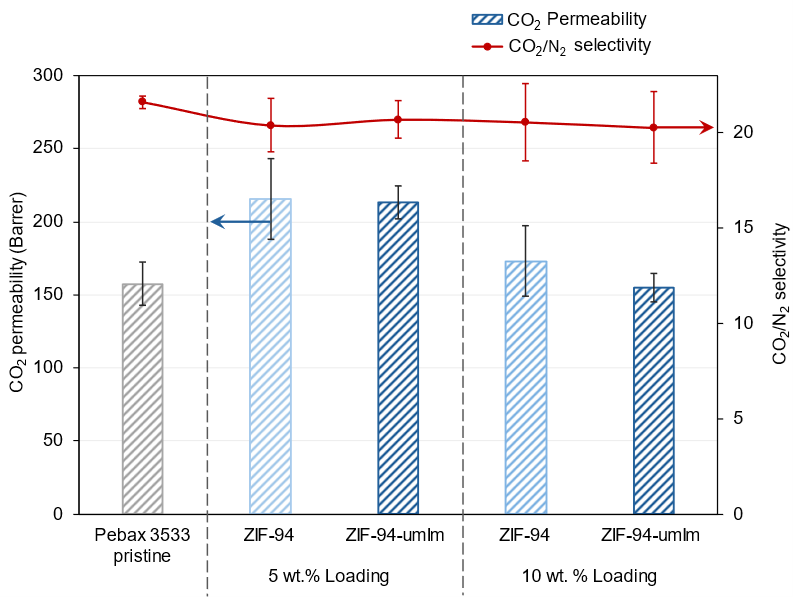


Figure S19. Gas separation performance of PEBAX^®^ 3533 and its MMMs with 5 and 10 wt.% loadings of ZIF-94 and ZIF-94-umIm for the separation of 15/85 CO_2_/N_2_.

**References Supporting Information:**

[1] A. B. Foster, M. Tamaddondar, J. M. Luque-Alled, W. J. Harrison, Z. Li, P. Gorgojo, P. M. Budd, *Macromolecules* **2020**, *53*, 569–583.

[2] N. Du, G. P. Robertson, J. Song, I. Pinnau, S. Thomas, M. D. Guiver, *Macromolecules* **2008**, *41*, 9656–9662.

[3] L. Paseta, M. Malankowska, C. Téllez, J. Coronas, *Materials Chemistry and Physics* **2023**, *295*, 127039.

[4] M. Pérez-Miana, J. M. Luque-Alled, M. Yahia, Á. Mayoral, J. Coronas, *J. Mater. Chem. A* **2024**, *12*, 10316–10328.

[5] M. Alberto, R. Bhavsar, J. M. Luque-Alled, E. Prestat, L. Gao, P. M. Budd, A. Vijayaraghavan, G. Szekely, S. M. Holmes, P. Gorgojo, *Journal of Membrane Science* **2018**, *565*, 390–401.

[6] A. F. Bushell, M. P. Attfield, C. R. Mason, P. M. Budd, Y. Yampolskii, L. Starannikova, A. Rebrov, F. Bazzarelli, P. Bernardo, J. Carolus Jansen, M. Lanč, K. Friess, V. Shantarovich, V. Gustov, V. Isaeva, *J. Membr. Sci.* **2013**, *427*, 48–62.

[7] J. Sánchez-Laínez, A. Pardillos-Ruiz, M. Carta, R. Malpass-Evans, N. B. McKeown, C. Téllez, J. Coronas, *Sep. Purif. Technol.* **2019**, *224*, 456–462.

[8] C. G. Bezzu, M. Carta, A. Tonkins, J. C. Jansen, P. Bernardo, F. Bazzarelli, N. B. McKeown, *Adv. Mater.* **2012**, *24*, 5930–5933.

[9] Y. Rogan, L. Starannikova, V. Ryzhikh, Y. Yampolskii, P. Bernardo, F. Bazzarelli, J. Carolus Jansen, N. B. McKeown, *Polym. Chem.* **2013**, *4*, 3813–3820.

[10] J. Zhang, H. Kang, J. Martin, S. Zhang, S. Thomas, T. C. Merkel, J. Jin, *Chem. Commun.* **2016**, *52*, 6553–6556.

[11] H. W. H. Lai, F. M. Benedetti, J. M. Ahn, A. M. Robinson, Y. Wang, I. Pinnau, Z. P. Smith, Y. Xia, *Science* **2022**, *375*, 1390–1392.

[12] L. Sun, W. Xu, H. Zhang, J. Chu, M. Wang, K. Song, W. Wu, J. Li, Y. Wang, I. Pinnau, X. Ma, *Angewandte Chemie International Edition* **n.d.**, *n/a*, e202420742.

[13] Z. Zhou, X. Cao, D. Lv, F. Cheng, *Separation and Purification Technology* **2024**, *339*, 126666.

[14] T. H. Lee, B. K. Lee, S. Y. Yoo, H. Lee, W.-N. Wu, Z. P. Smith, H. B. Park, *Nat Commun* **2023**, *14*, 8330.

[15] G. Dai, Q. Zhang, S. Xiong, L. Deng, Z. Gao, A. Chen, X. Li, C. Pan, J. Tang, G. Yu, *Journal of Membrane Science* **2023**, *676*, 121561.

[16] C. Ye, X. Wu, H. Wu, L. Yang, Y. Ren, Y. Wu, Y. Liu, Z. Guo, R. Zhao, Z. Jiang, *Chemical Engineering Science* **2020**, *216*, 115497.

[17] A. Fuoco, M. R. Khdhayyer, M. P. Attfield, E. Esposito, J. C. Jansen, P. M. Budd, *Membranes* **2017**, *7*, 7.

[18] X. Wu, W. Liu, H. Wu, X. Zong, L. Yang, Y. Wu, Y. Ren, C. Shi, S. Wang, Z. Jiang, *J. Membr. Sci.* **2018**, *548*, 309–318.

[19] J. Han, L. Bai, H. Jiang, S. Zeng, B. Yang, Y. Bai, X. Zhang, *Ind. Eng. Chem. Res.* **2021**, *60*, 593–603.

[20] L. Hao, K.-S. Liao, T.-S. Chung, *Journal of Materials Chemistry A* **2015**, *3*, 17273–17281.

[21] K. Xie, Q. Fu, G. G. Qiao, P. A. Webley, *Journal of Membrane Science* **2019**, *572*, 38–60.

[22] B. Qiu, M. Yu, J. M. Luque-Alled, S. Ding, A. B. Foster, P. M. Budd, X. Fan, P. Gorgojo, *Angewandte Chemie* **2024**, *136*, e202316356.
